# Supplementary material for: Warhead biosynthesis and the origin of structural diversity in hydroxamate metalloproteinase inhibitors
Source: Nat Commun. 2017 Dec 6;8:1965. doi: 10.1038/s41467-017-01975-6 (PMC5719088; doi:10.1038/s41467-017-01975-6)
Supplement: Supplementary file 1 — Supplementary Information [file 41467_2017_1975_MOESM1_ESM.pdf]

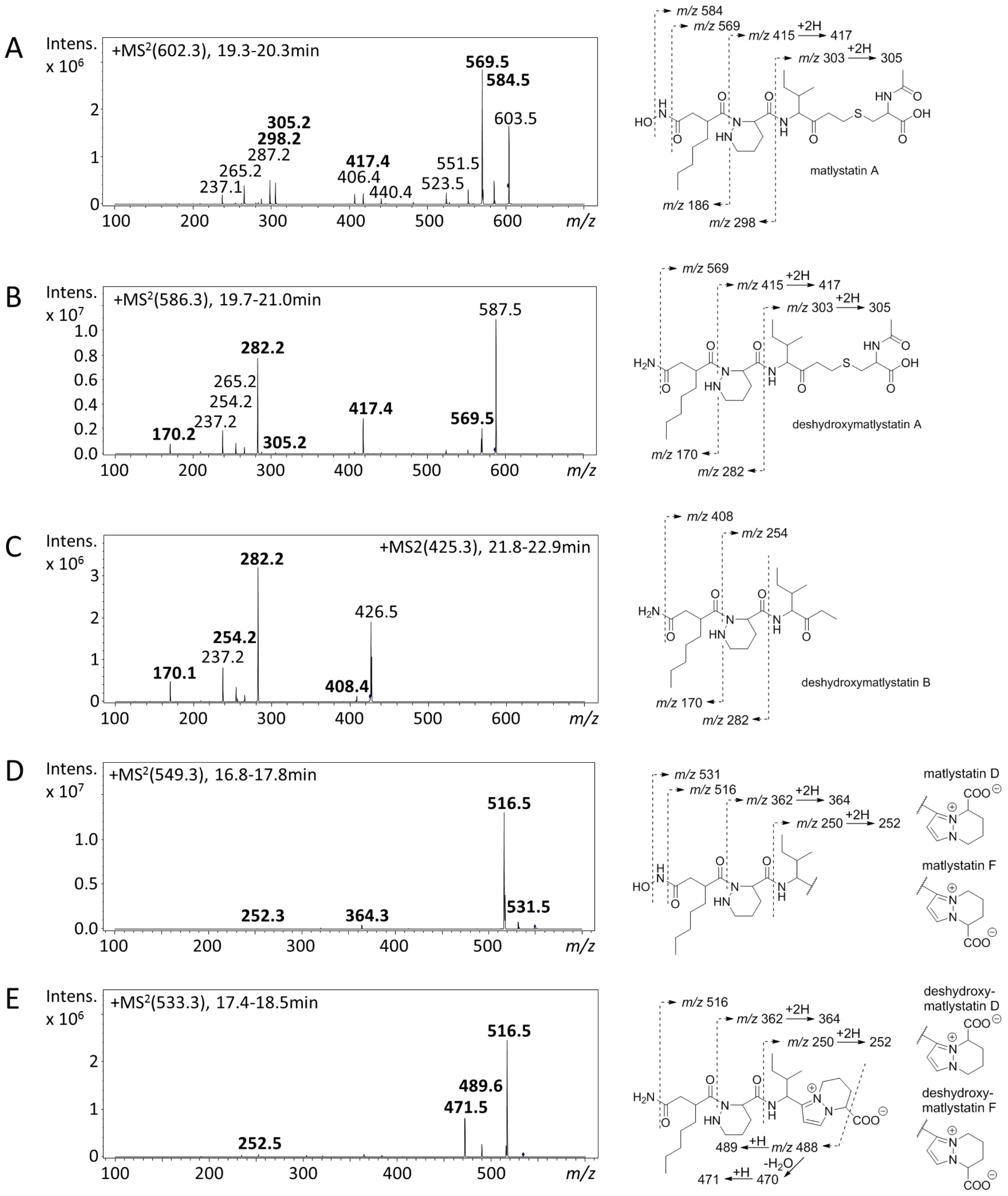
A
Intens. x 10^6
+MS²(602.3), 19.3-20.3min
569.5
584.5
603.5
305.2
298.2
287.2
265.2
237.1
417.4
406.4
440.4
551.5
523.5
m/z 584
m/z 569
m/z 415 +2H 417
m/z 303 +2H 305
m/z 186
m/z 298
matlystatin A
B
Intens. x 10^7
+MS²(586.3), 19.7-21.0min
587.5
282.2
265.2
254.2
237.2
170.2
305.2
417.4
569.5
m/z 569
m/z 415 +2H 417
m/z 303 +2H 305
m/z 170
m/z 282
deshydroxymatlystatin A
C
Intens. x 10^6
+MS2(425.3), 21.8-22.9min
282.2
426.5
254.2
237.2
170.1
408.4
m/z 408
m/z 254
m/z 170
m/z 282
deshydroxymatlystatin B
D
Intens. x 10^7
+MS²(549.3), 16.8-17.8min
516.5
531.5
252.3
364.3
m/z 531
m/z 516
m/z 362 +2H 364
m/z 250 +2H 252
matlystatin D
matlystatin F
E
Intens. x 10^6
+MS²(533.3), 17.4-18.5min
516.5
489.6
471.5
252.5
m/z 516
m/z 362 +2H 364
m/z 250 +2H 252
489 +H m/z 488
-H2O
471 +H 470
deshydroxy-matlystatin D
deshydroxy-matlystatin F
m/z

F

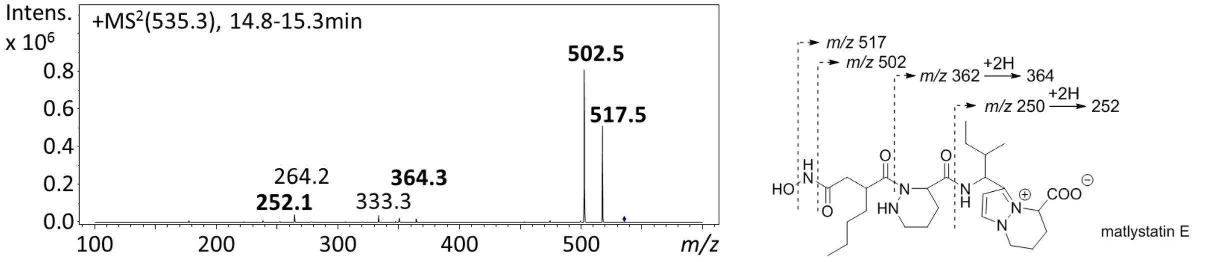

G

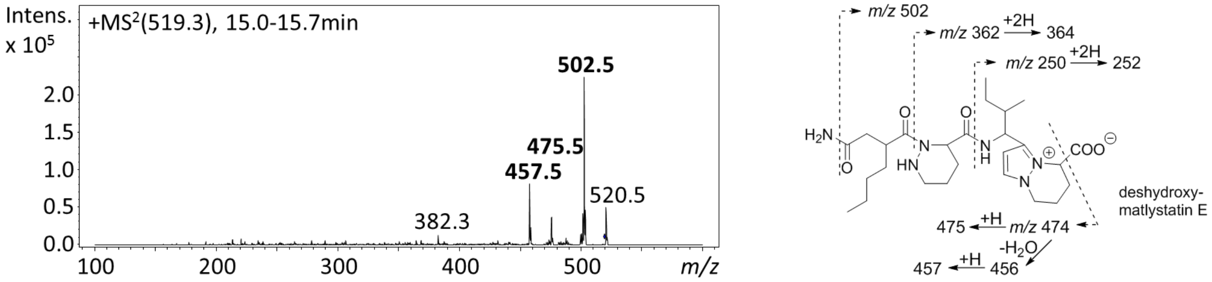

Supplementary Figure 1. Proposed fragmentation scheme and MS^2 spectrum of matlystatins as produced in *Actinomadura atramentaria* DSM 43919. Assigned fragments are indicated as bold. Data were acquired via method 1.

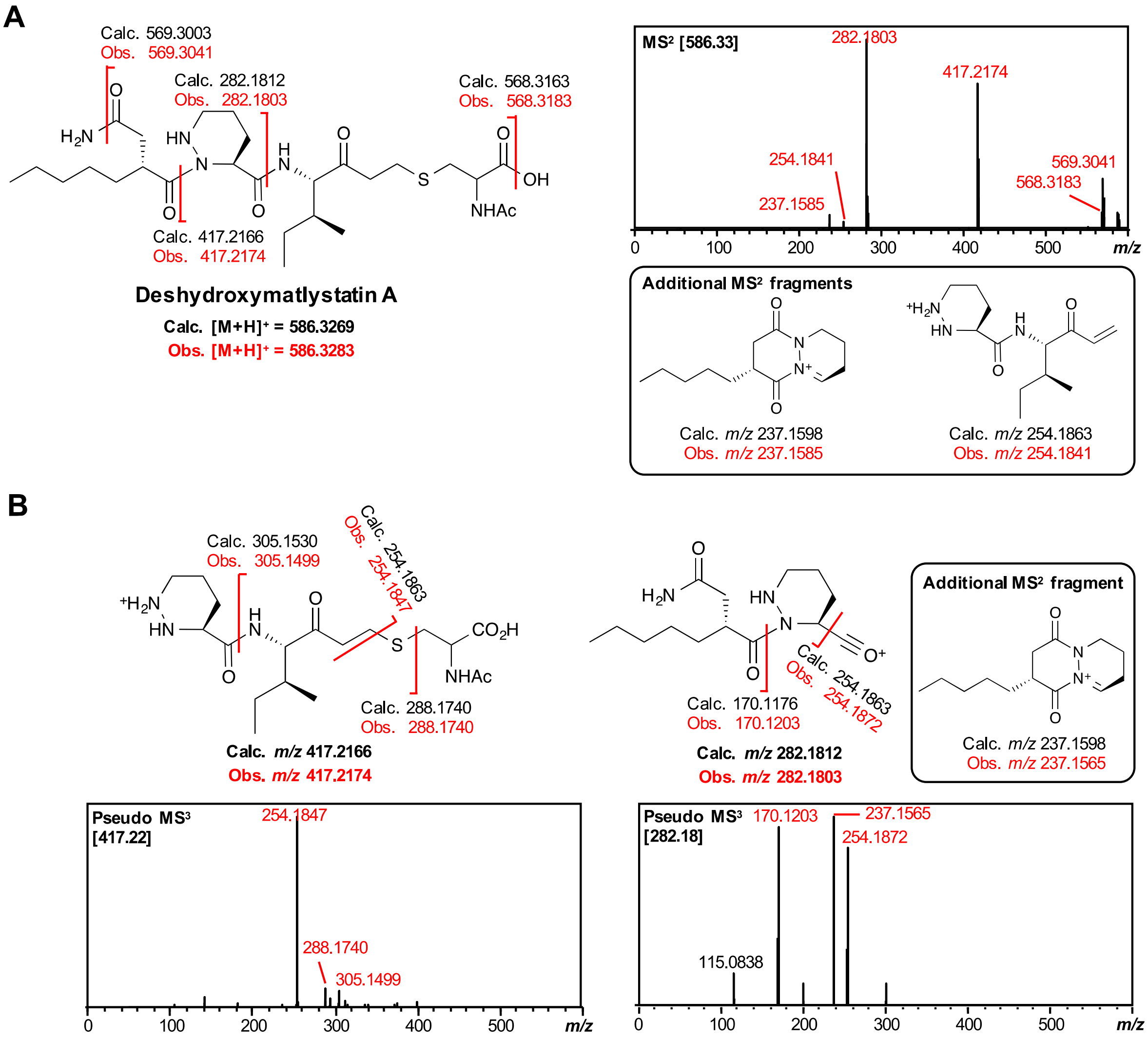

Supplementary Figure 2. MS^2 and pseudo MS^3 analysis of deshydroxymatlystatin A. Data were acquired via method 2.

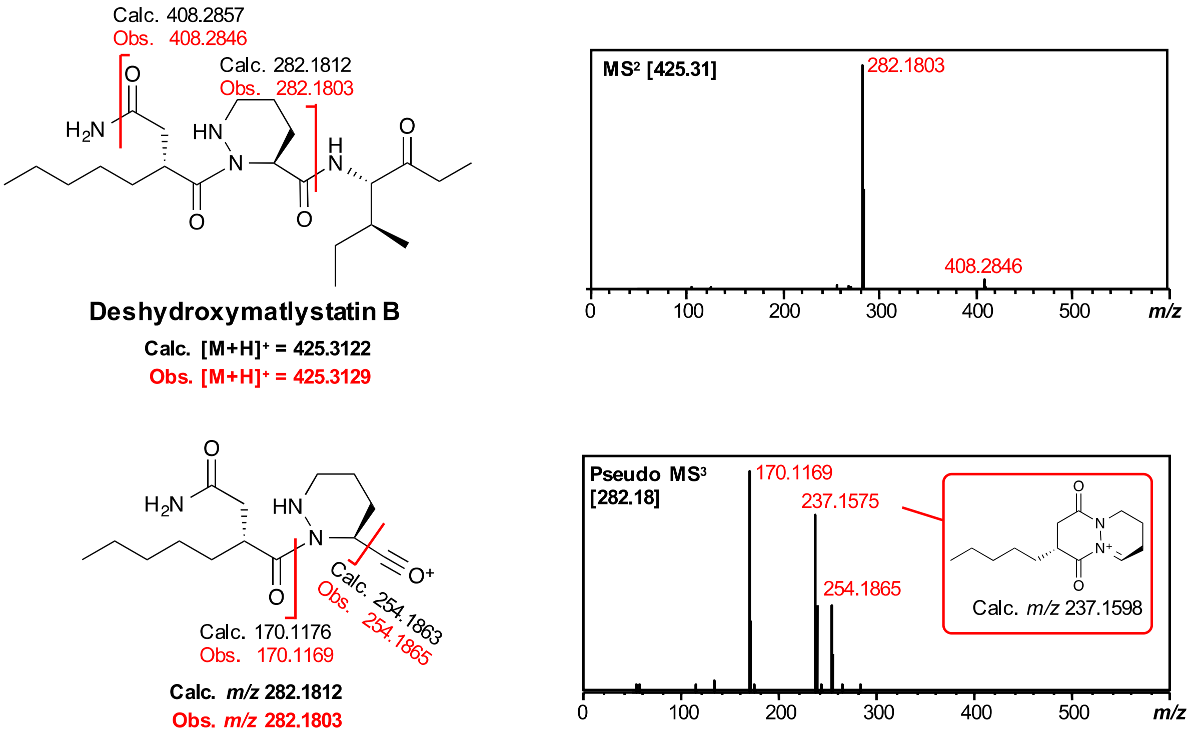

Supplementary Figure 3. MS^2 and pseudo MS^3 analysis of deshydroxymatlystatin B. Data were acquired via method 2.

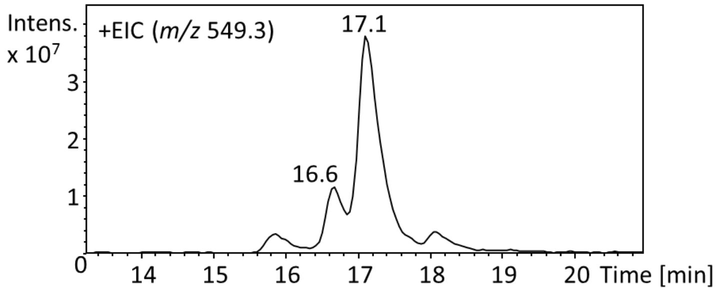

Supplementary Figure 4. Peak splitting in an extracted ion chromatogram from LC-MS analysis of *A. atramentaria* culture extracts for *m/z* 549.3 $[M+H]^+$ (matlystatin D and F isomers). Data were acquired via method 1.

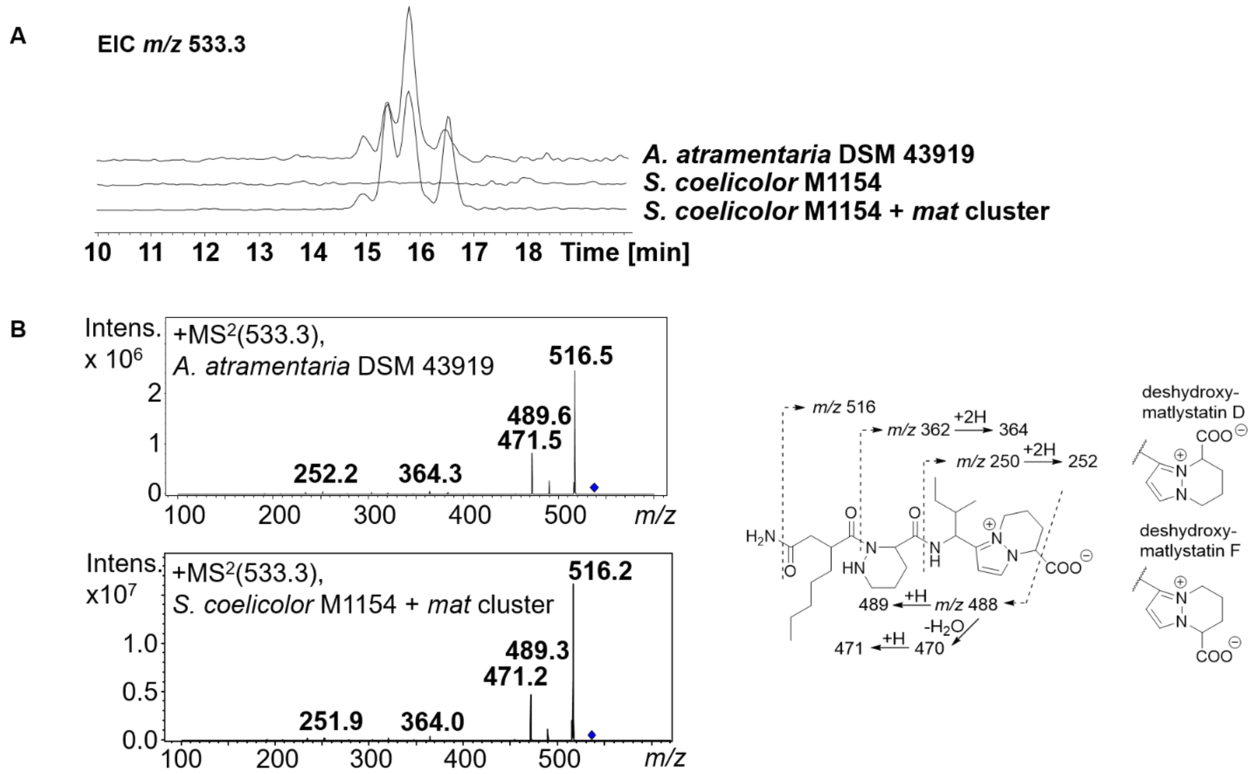

Supplementary Figure 5. LC-MS and MS^2 analysis of heterologously produced deshydroxymatlystatin D and F. Comparison of extracted ion chromatograms (EIC; A) and MS^2 spectra (B) from the wild type and the heterologous producer. Data were acquired via method 1.

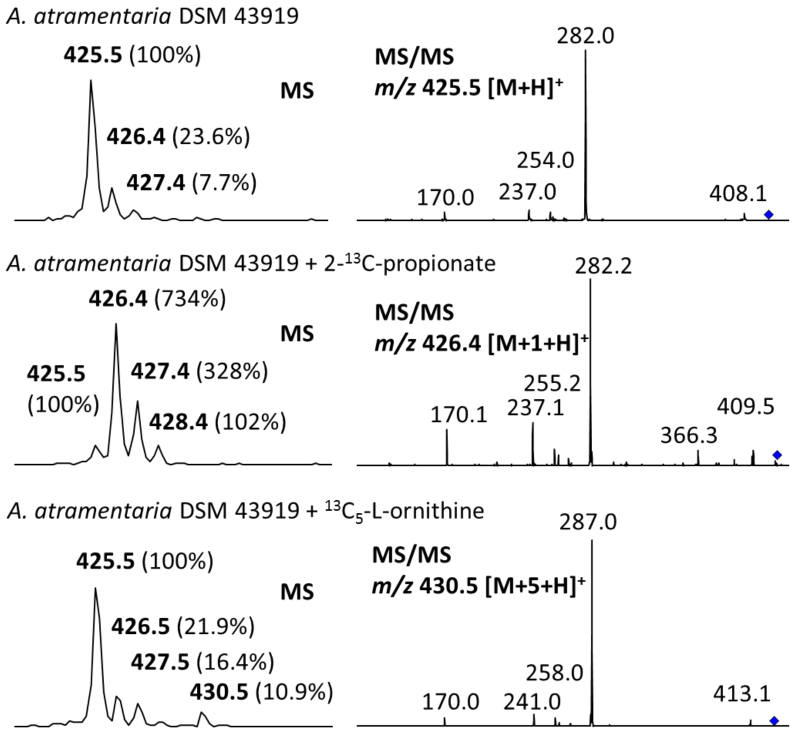

Supplementary Figure 6. MS analysis of feeding studies with [2-^{13}C]propionate and [U-$^{13}C_5$]-L-ornithine. Isotopic distribution and MS^2 spectrum of deshydroxymatlystatin B. Data were acquired via method 1.

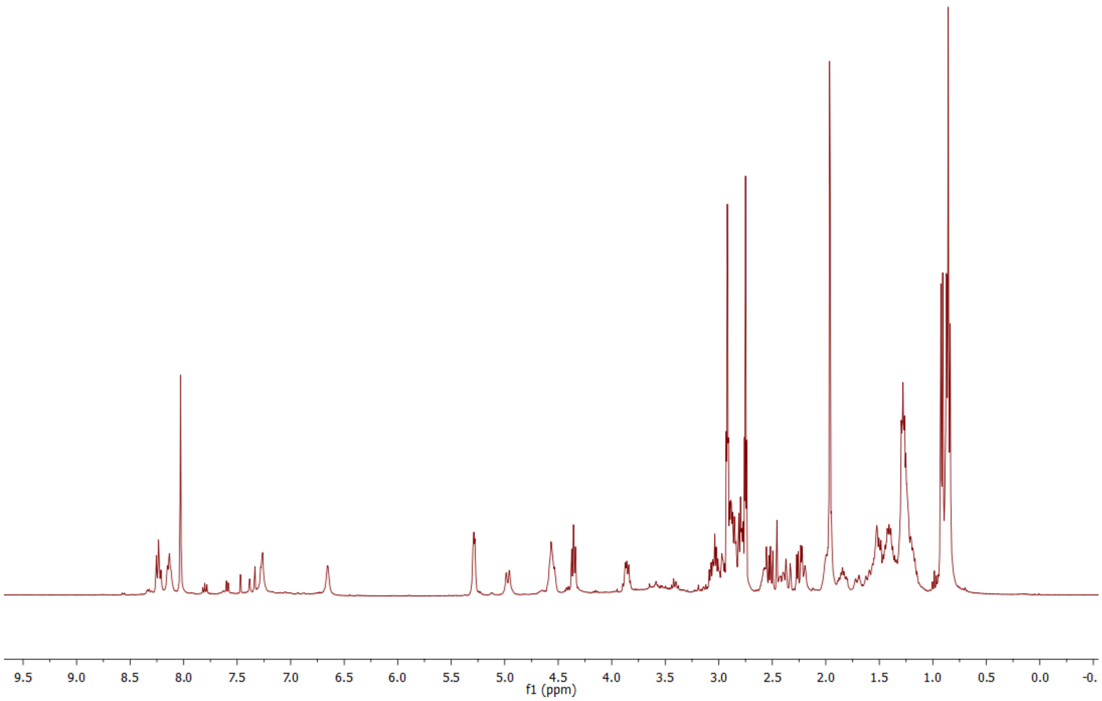

Supplementary Figure 7. 400 MHz ^{1}H NMR spectrum of deshydroxymatlystatin A (**1a**) in d_7-DMF.

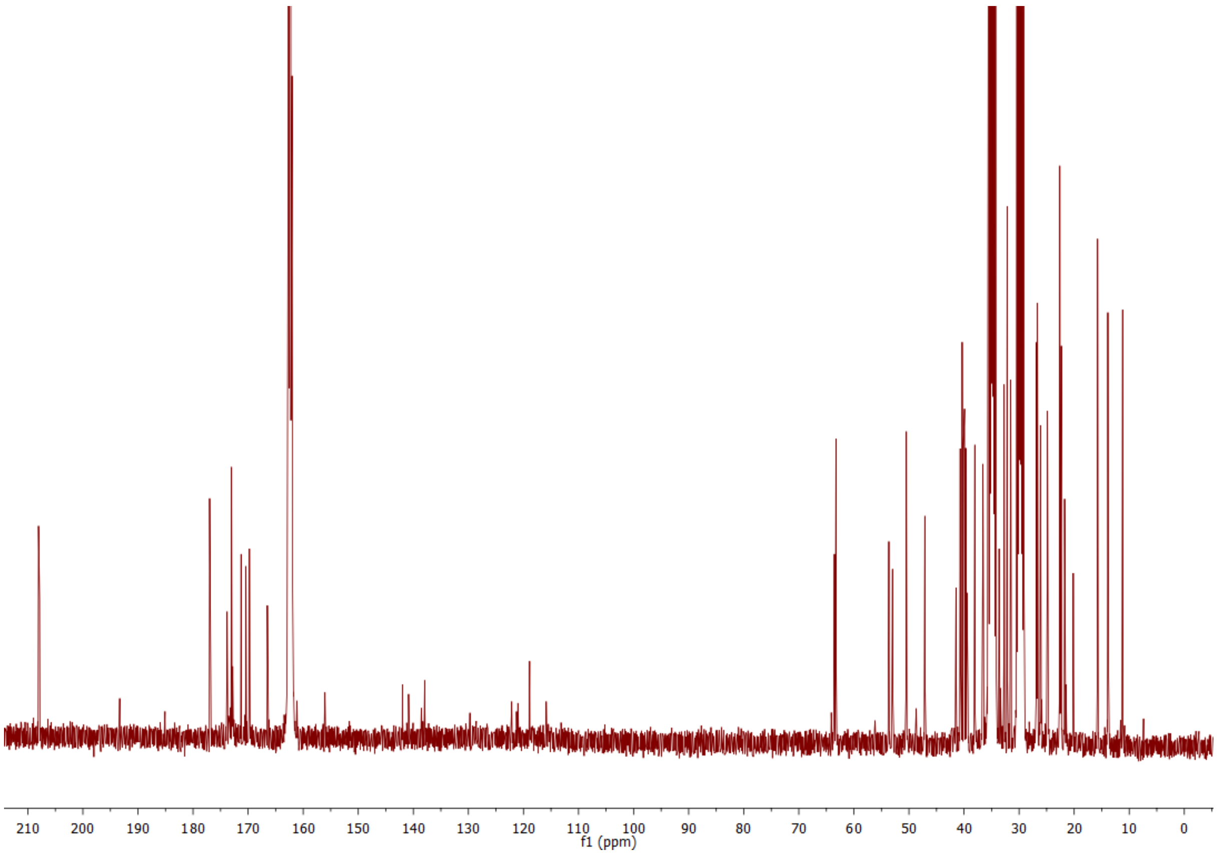

Supplementary Figure 8. 101 MHz ^{13}C NMR spectrum of **1a** in d_7-DMF.

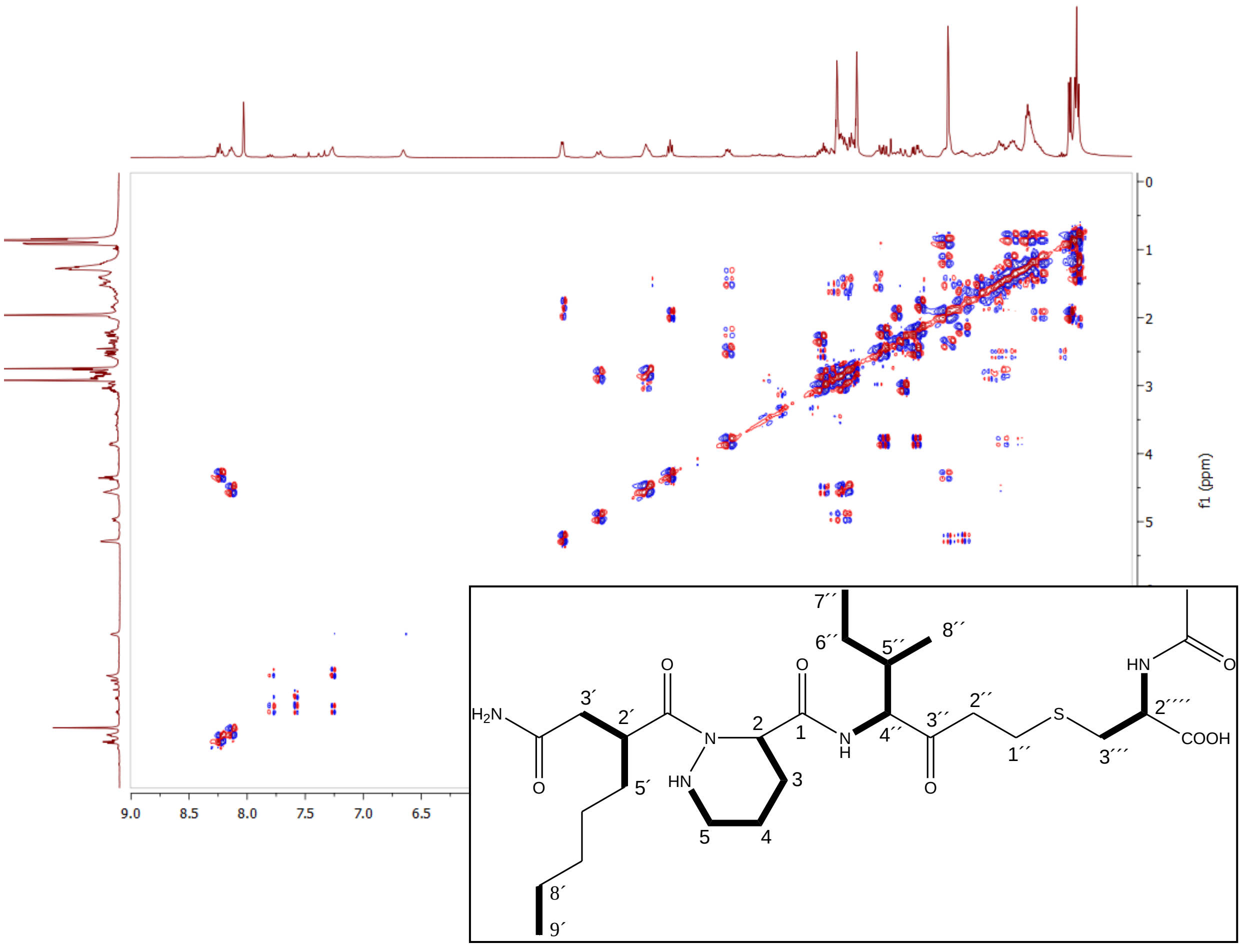

Supplementary Figure 9. 400 MHz ^{1}H-^{1}H-DQF-COSY NMR spectrum of **1a** in *d*$_7$-DMF. Bold lines in the insert visualize the observed ^{1}H-^{1}H-COSY-correlations.

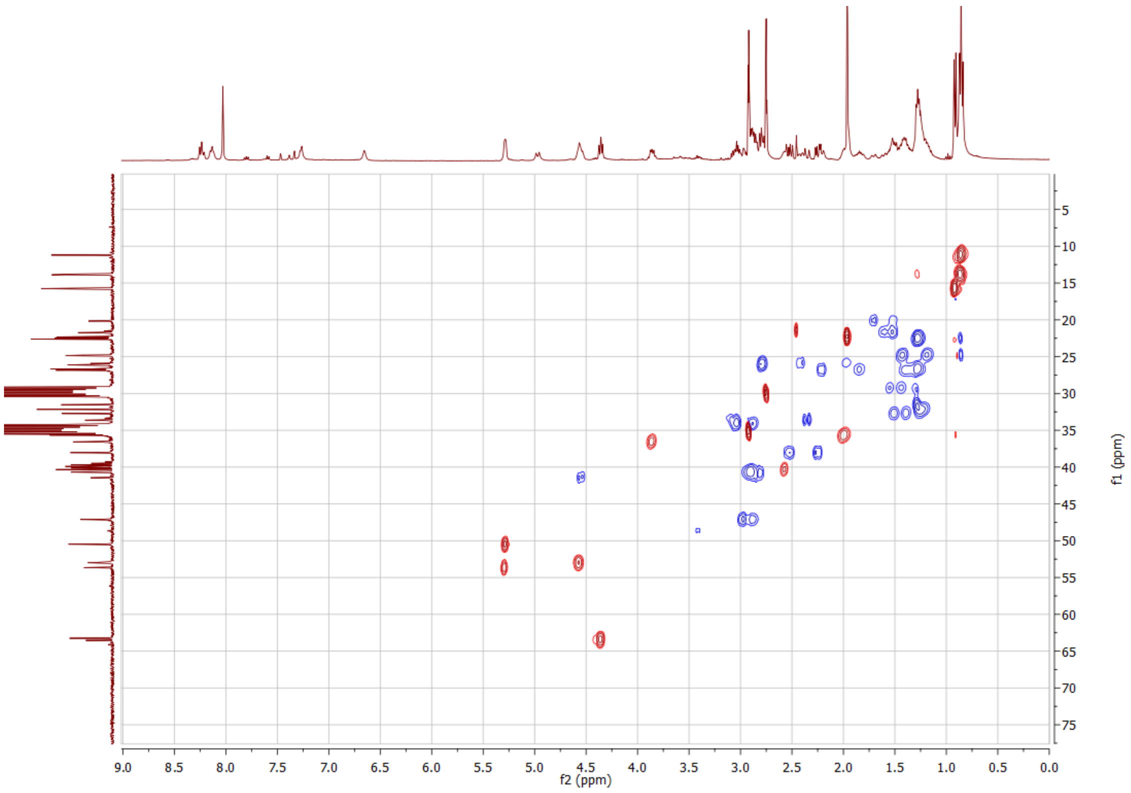

Supplementary Figure 10. 400 MHz ^{1}H-^{13}C-multiplicity-edited-HSQC NMR spectrum of **1a** in d_7-DMF.

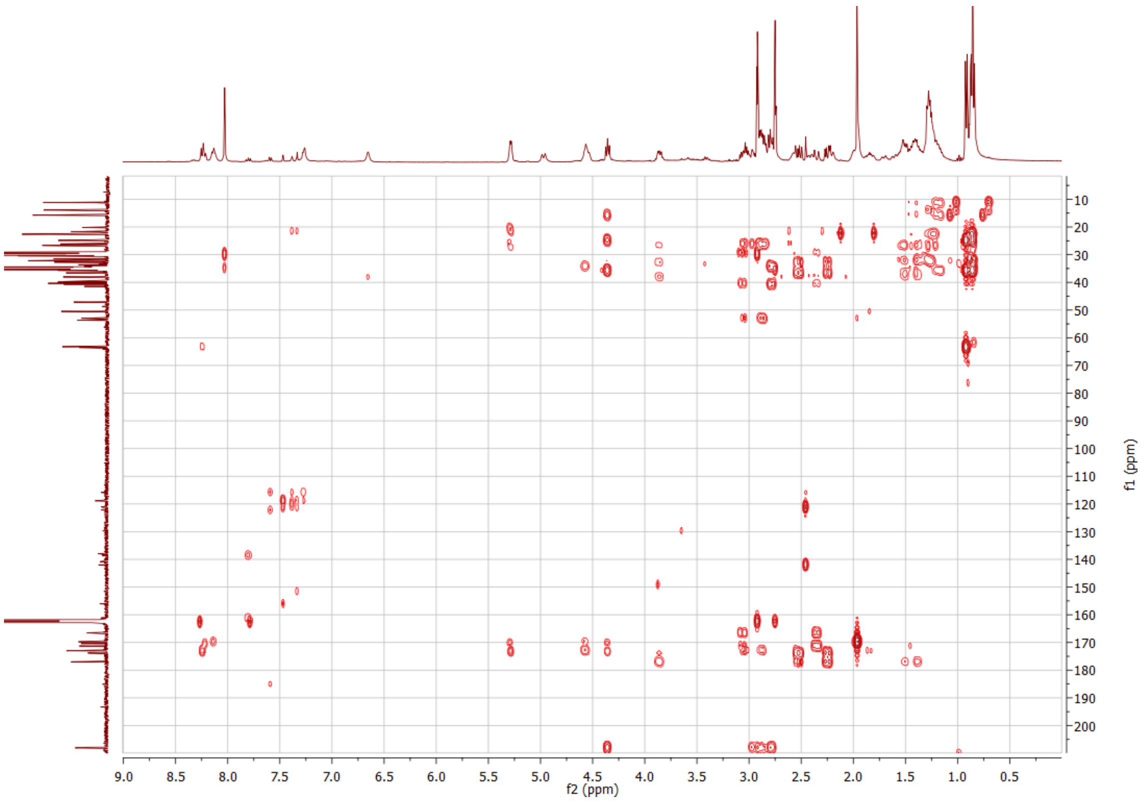

Supplementary Figure 11. 400 MHz ^{1}H-^{13}C-HMBC NMR spectrum of **1a** in d_7-DMF.

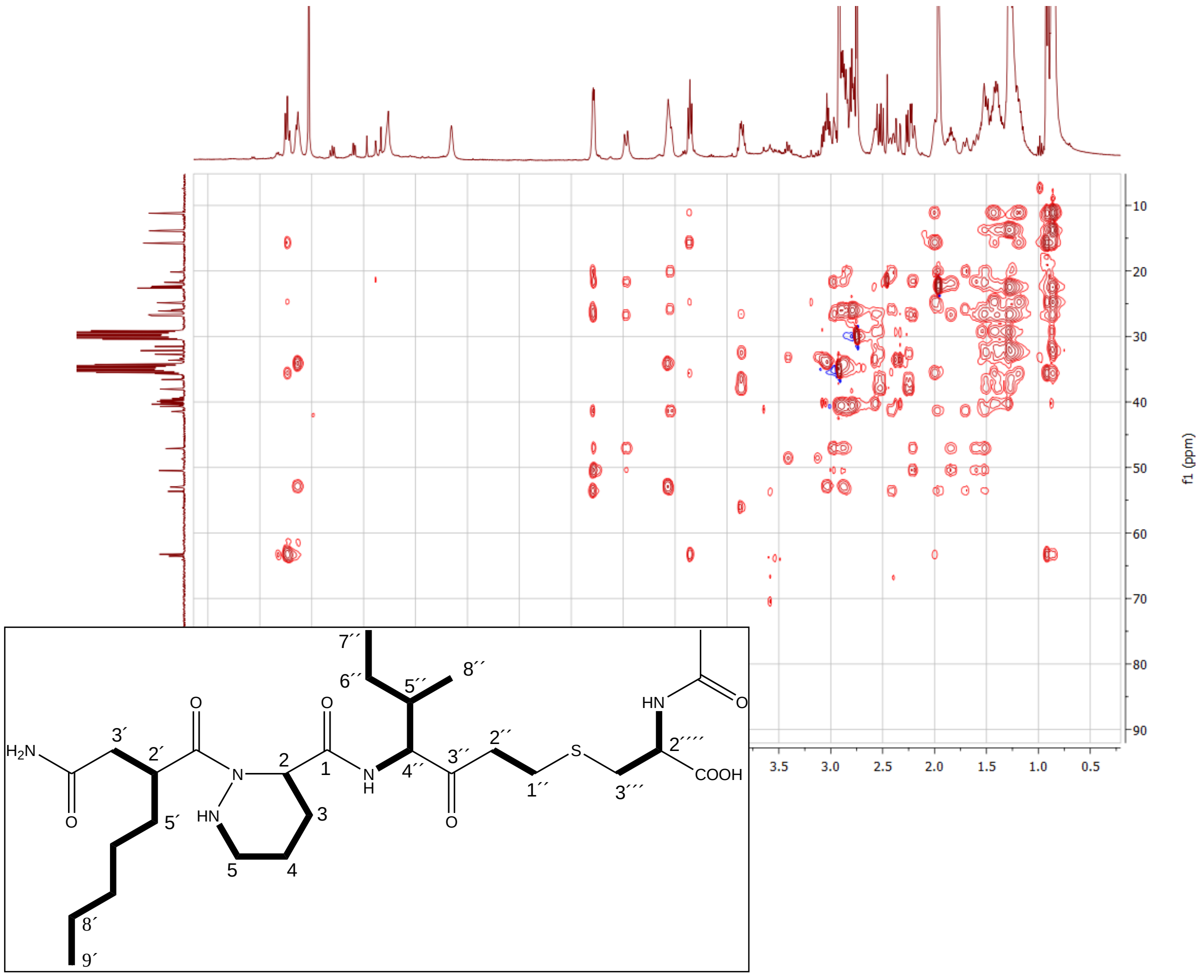

Supplementary Figure 12. 400 MHz ^{1}H-^{13}C-HSQC-TOCSY NMR spectrum of **1a** in *d*$_7$-DMF. Bold lines in the insert visualize the observed cross-correlations.

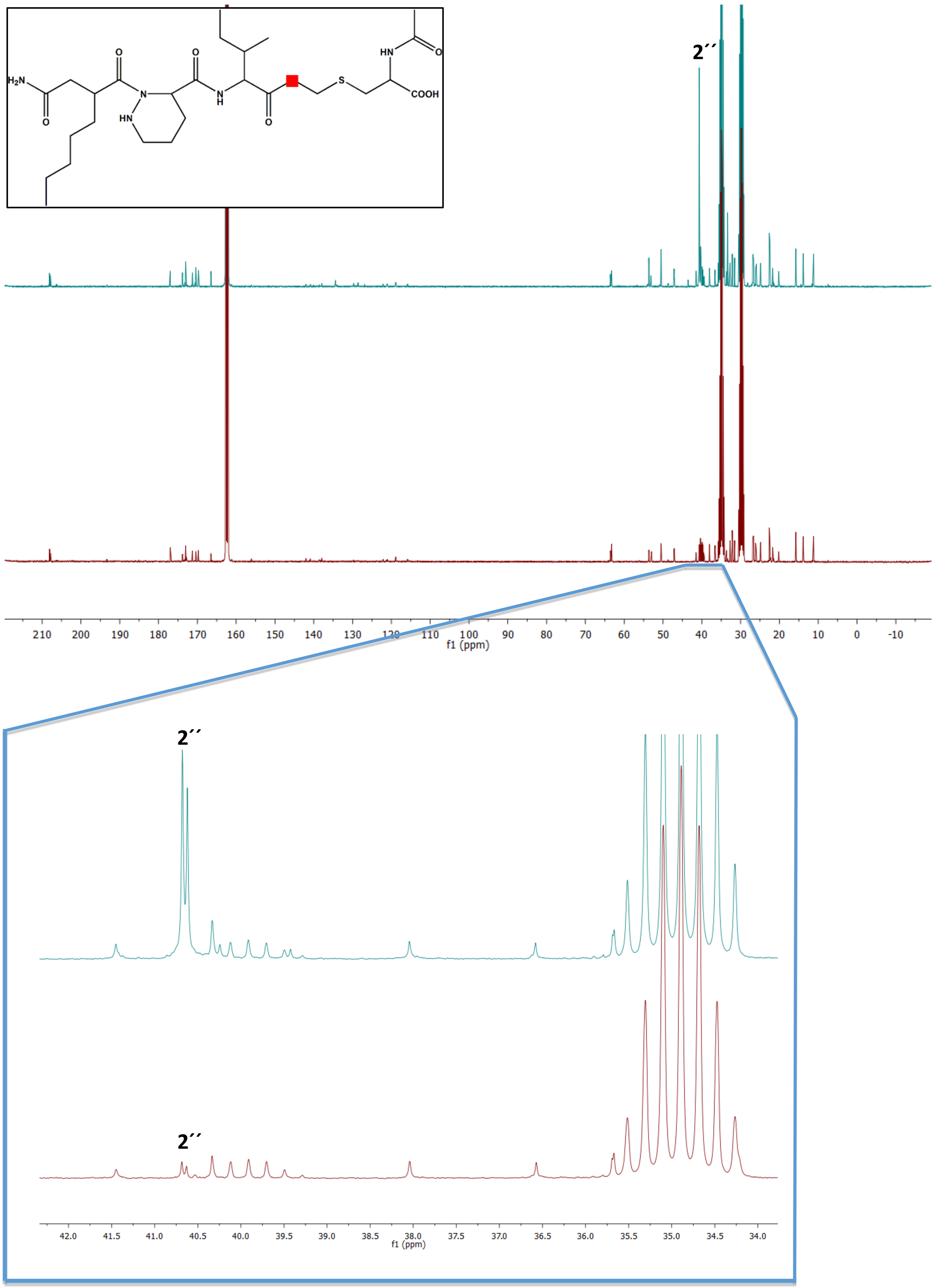

H2N
O
O
N
HN
O
N
H
O
S
HN
O
COOH
2´´
2´´
2´´
f1 (ppm)
f1 (ppm)

Supplementary Figure 13. 101 MHz ^{13}C NMR spectra of **1a** in d_7-DMF, labeled with [2-^{13}C]propionate (blue top spectrum) and in natural abundance (red bottom spectrum). The concentration was adjusted in both samples to 33 mg/mL. Bold number in the spectrum and red box in the insert indicate the significantly enriched carbon atom. Blue box shows an expansion (34-42 ppm) of the ^{13}C NMR spectrum.

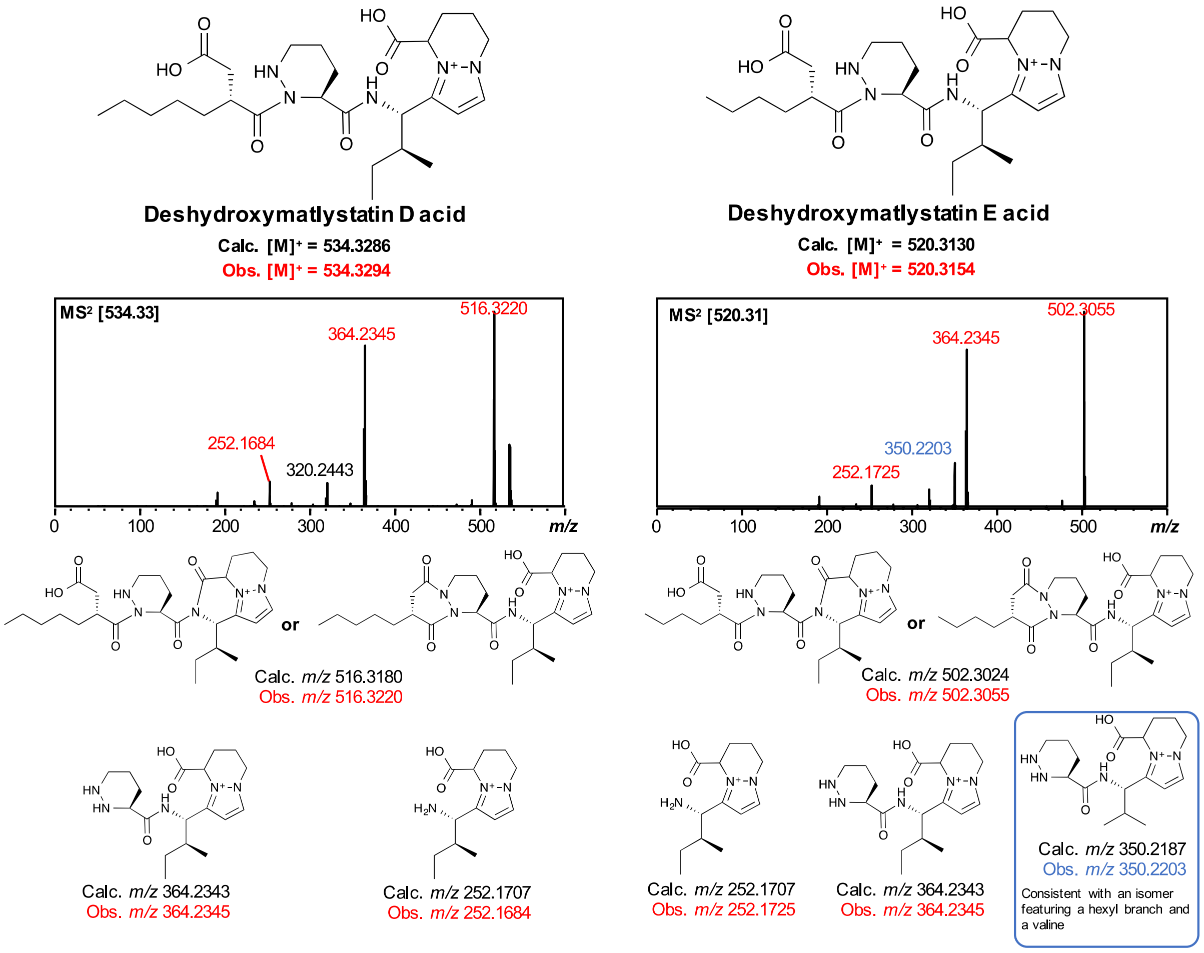

Supplementary Figure 14. MS^2 analysis of carboxylic acid versions of deshydroxymatlystatins D and E.

Data were acquired via method 2.

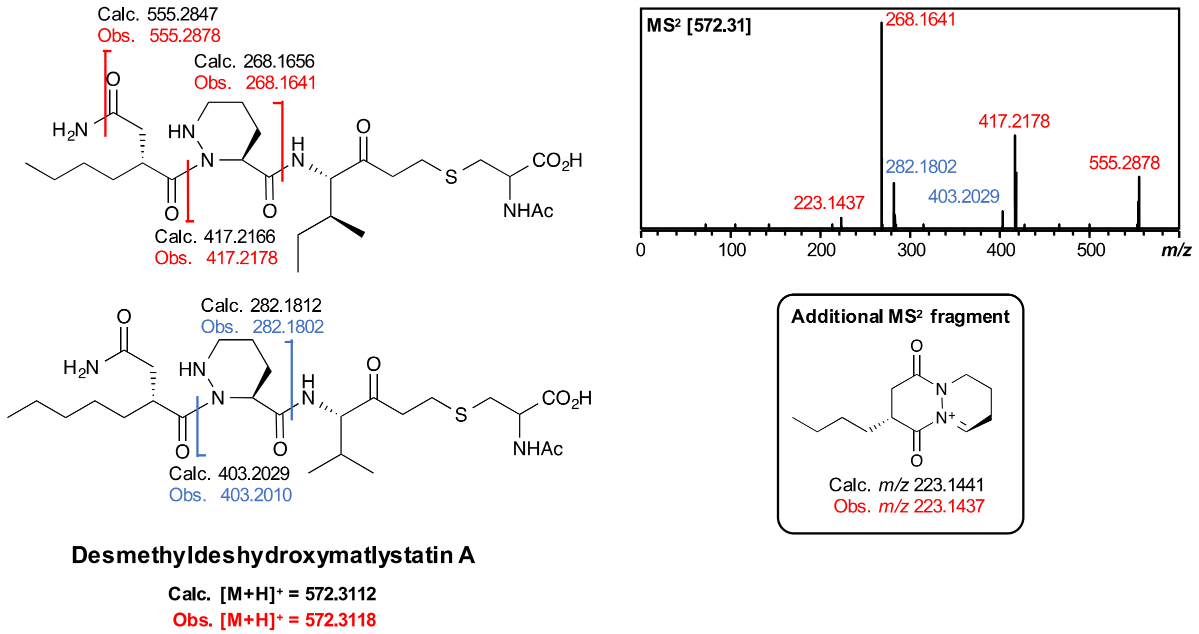

Supplementary Figure 15. MS^2 analysis of desmethyldeshydroxymatlystatin A. Data were acquired via method 2.

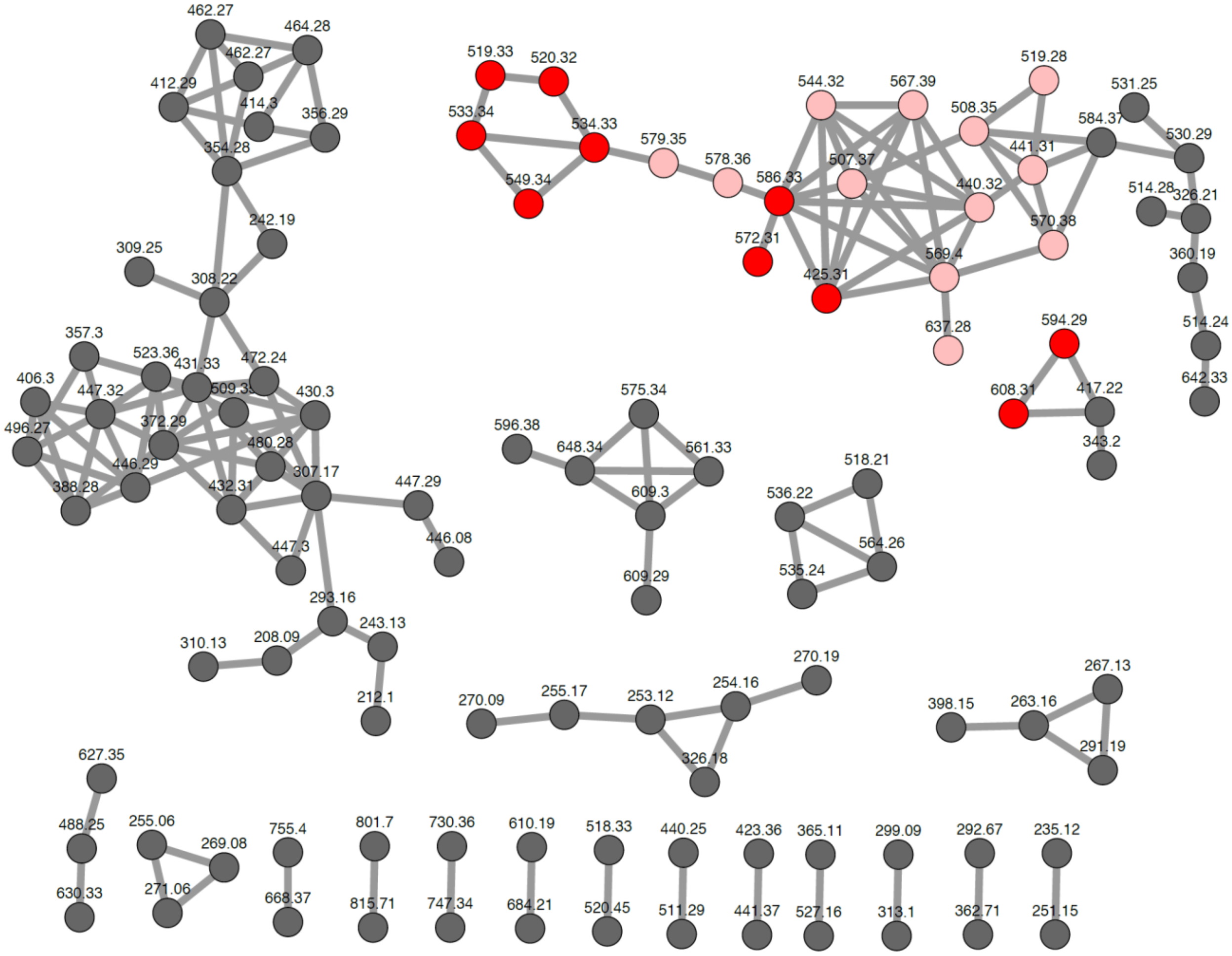

Supplementary Figure 16. Total mass spectral network using the combined MS2 data from A. atramentaria DSM 43919 fermentations in MPM, BPM and TSB. The network has been filtered based on criteria reported in the methods section and node labels indicate the m/z values for each molecule in the network. See Supplementary Data 1 (matlystatin_network.cys) for this network, as well as a network relating to the nodes reported in Figure 5. Known matlystatin congeners are highlighted in bright red and other matlystatin-like molecules reported in this study are highlighted in pale red. Other molecules belonging to matlystatin-containing networks could not be confidently assigned based on MS^n data. The small network with two matlystatins highlighted contains the sodium adducts of deshydroxymatlystatin A and a related compound with a shorter acyl chain (- 14 Da).

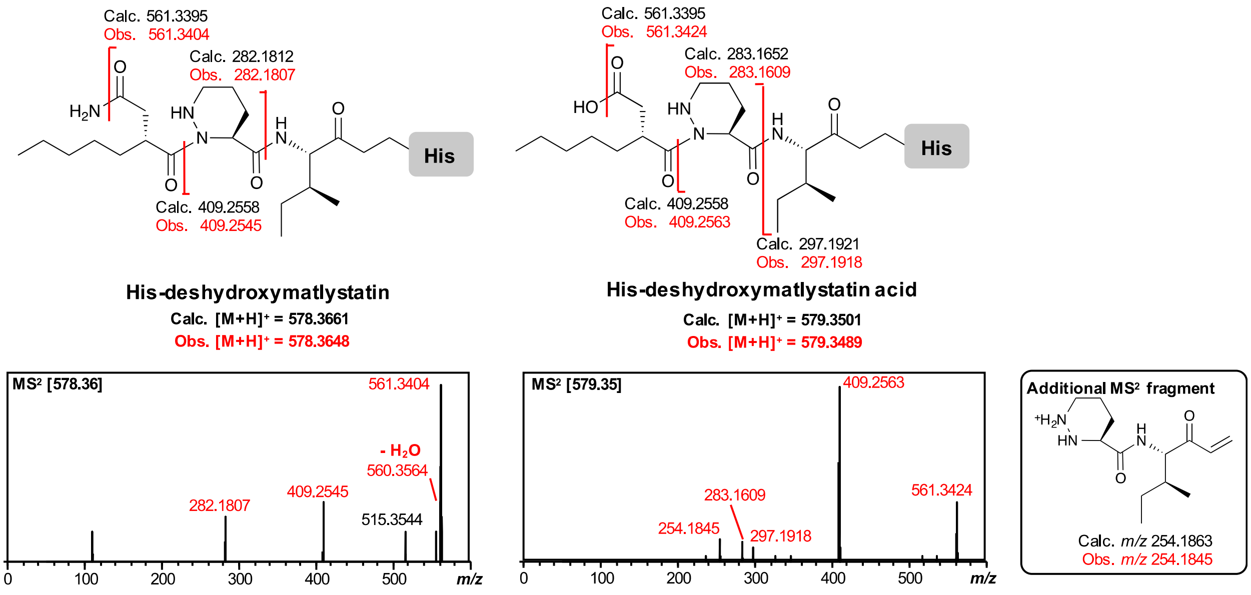

Supplementary Figure 17. MS^2 analysis of putative histidine adducts identified by mass spectral networking. Data were acquired via method 2.

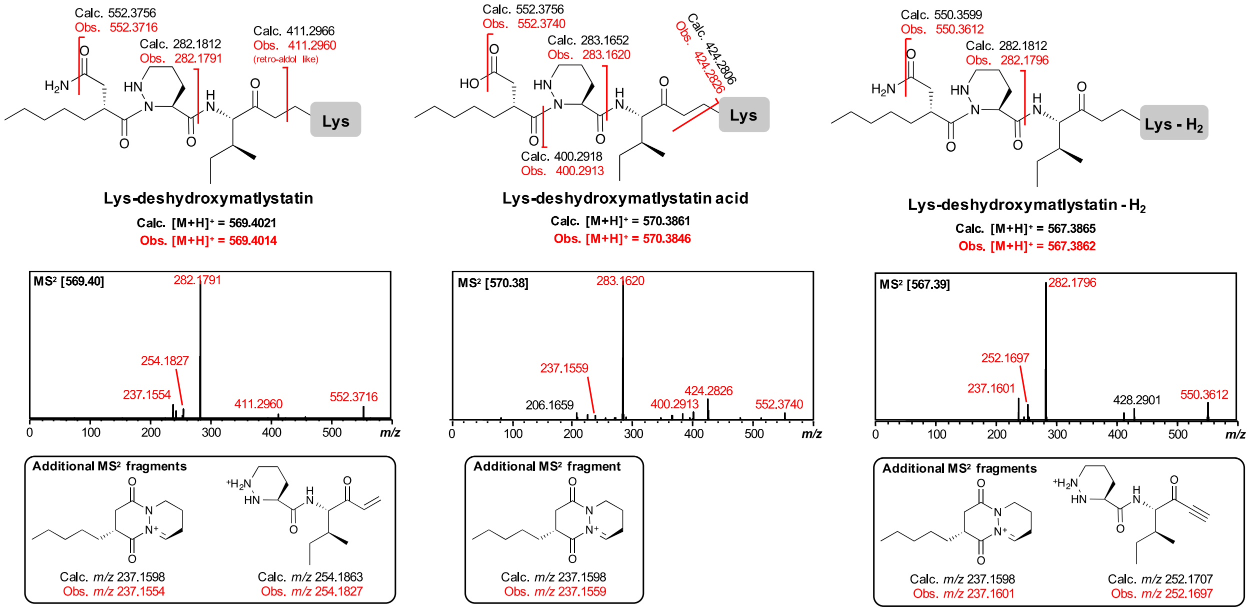

Supplementary Figure 18. MS^2 analysis of putative lysine adducts identified by mass spectral networking.

Data were acquired via method 2.

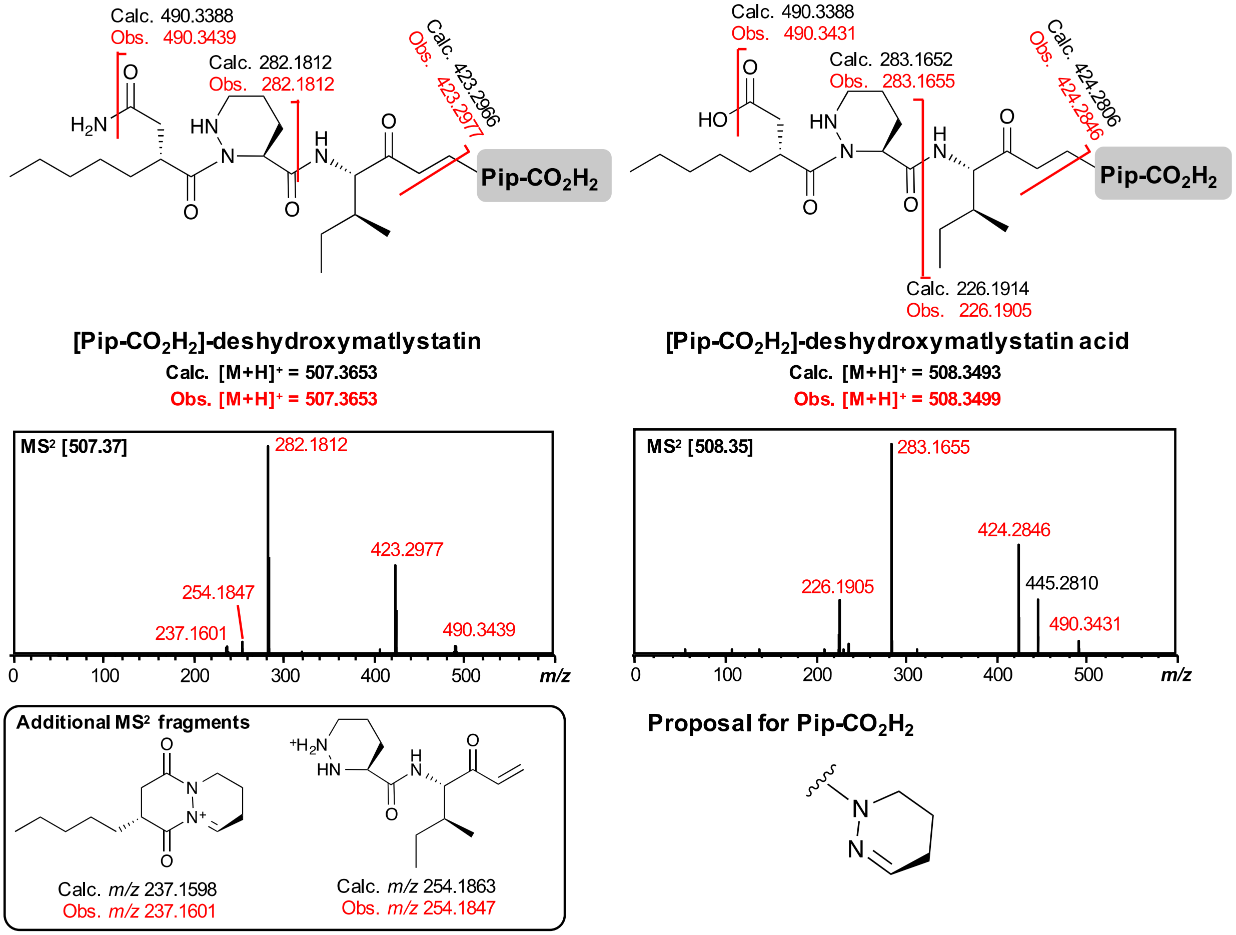

Supplementary Figure 19. MS[2] analysis of putative [Pip-CO_2H_2] adducts identified by mass spectral networking. Data were acquired via method 2.

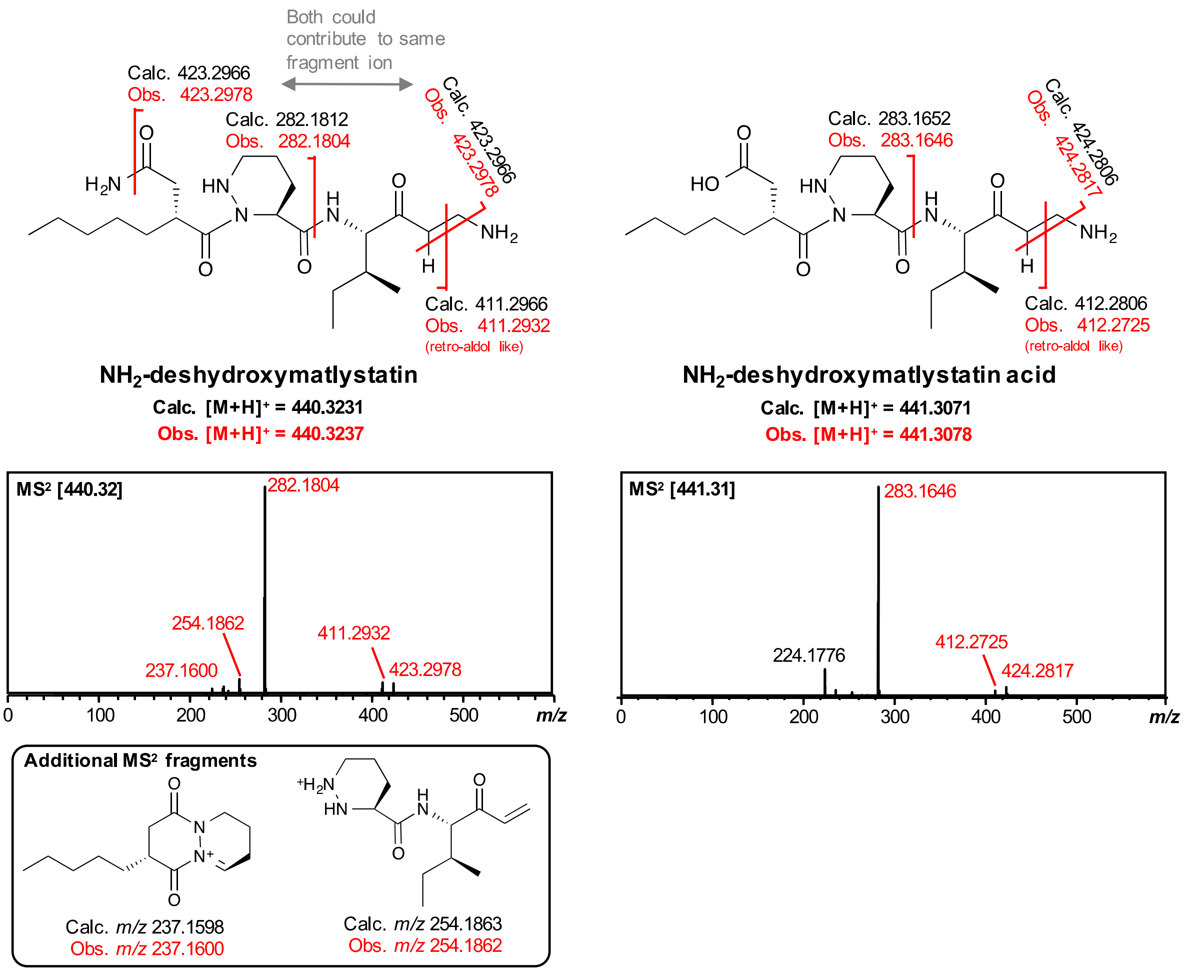

Supplementary Figure 20. MS^2 analysis of putative NH_3 adducts identified by mass spectral networking.

Data were acquired via method 2.

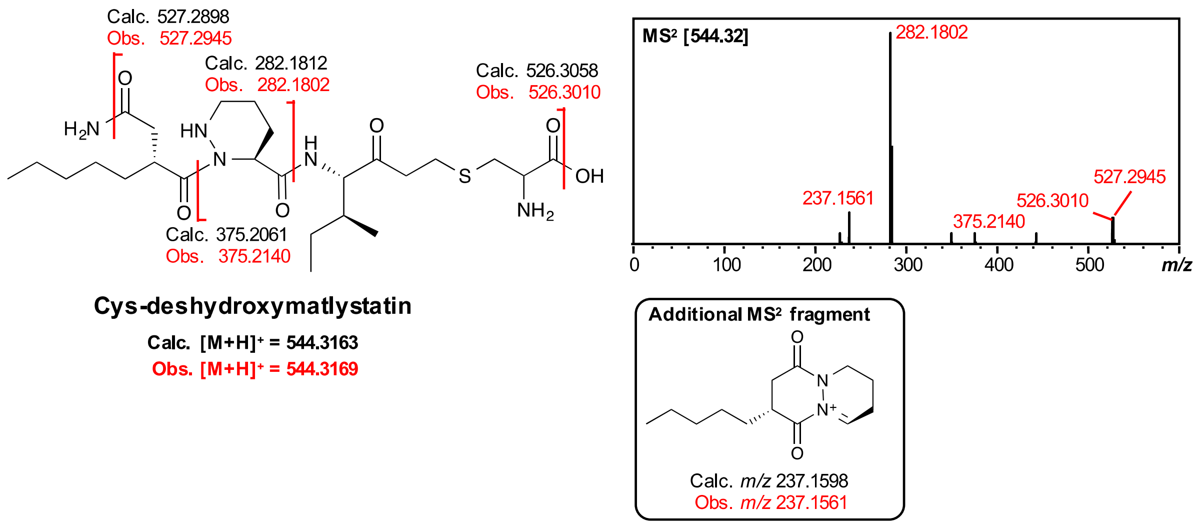

Supplementary Figure 21. MS^2 analysis of putative cysteine adduct identified by mass spectral networking. Proposed structure features conjugate addition from sulfur, but the reaction from the amino group is possible. Data were acquired via method 2.

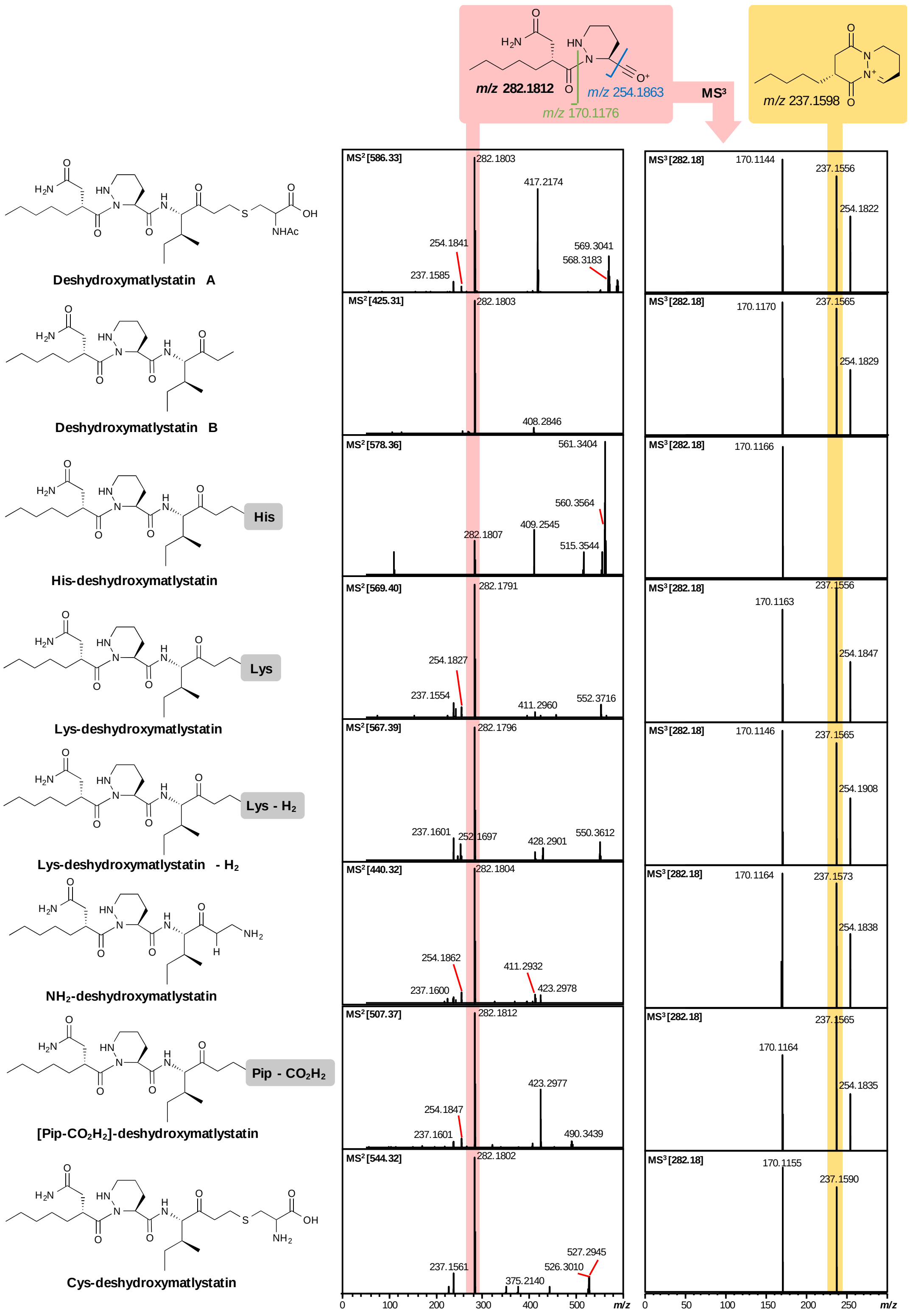

Supplementary Figure 22. MS^n analysis of known and new deshydroxymatlystatin congeners identified by mass spectral networking showing common MS^n patterns. Data were acquired via method 2.

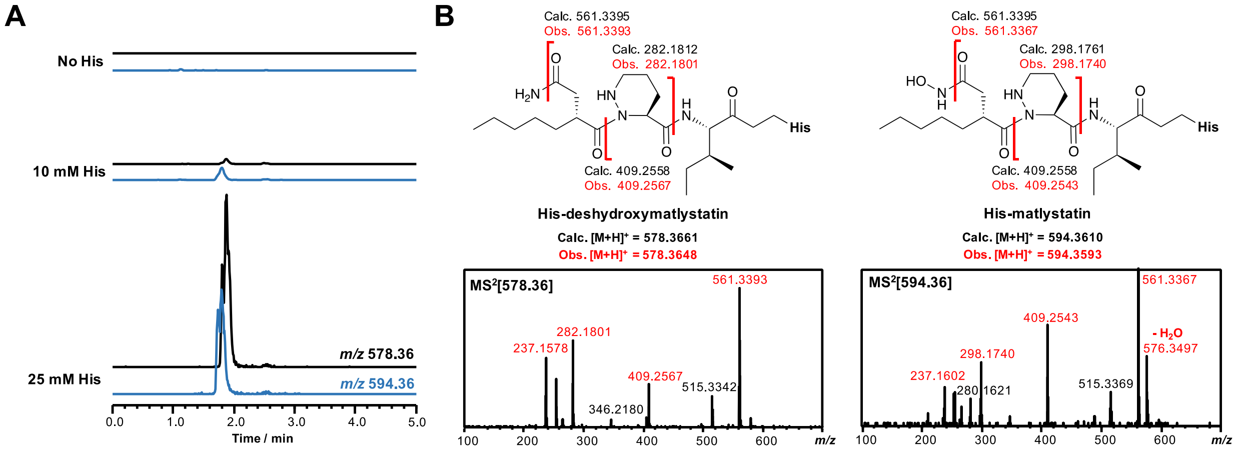

Supplementary Figure 23. Feeding of L-histidine to *A. atramentaria*. (A) Production of putative His-deshydroxymatlystatin and His-matlystatin congeners following feeding of histidine to production cultures in MPM, a medium that does not naturally support the production of these compounds. (B) MS^2 analysis of the compounds with *m/z* 578.36 and *m/z* 594.36. The identity of *m/z* 237.16 is indicated in Fig. S22. Data were acquired via method 2.

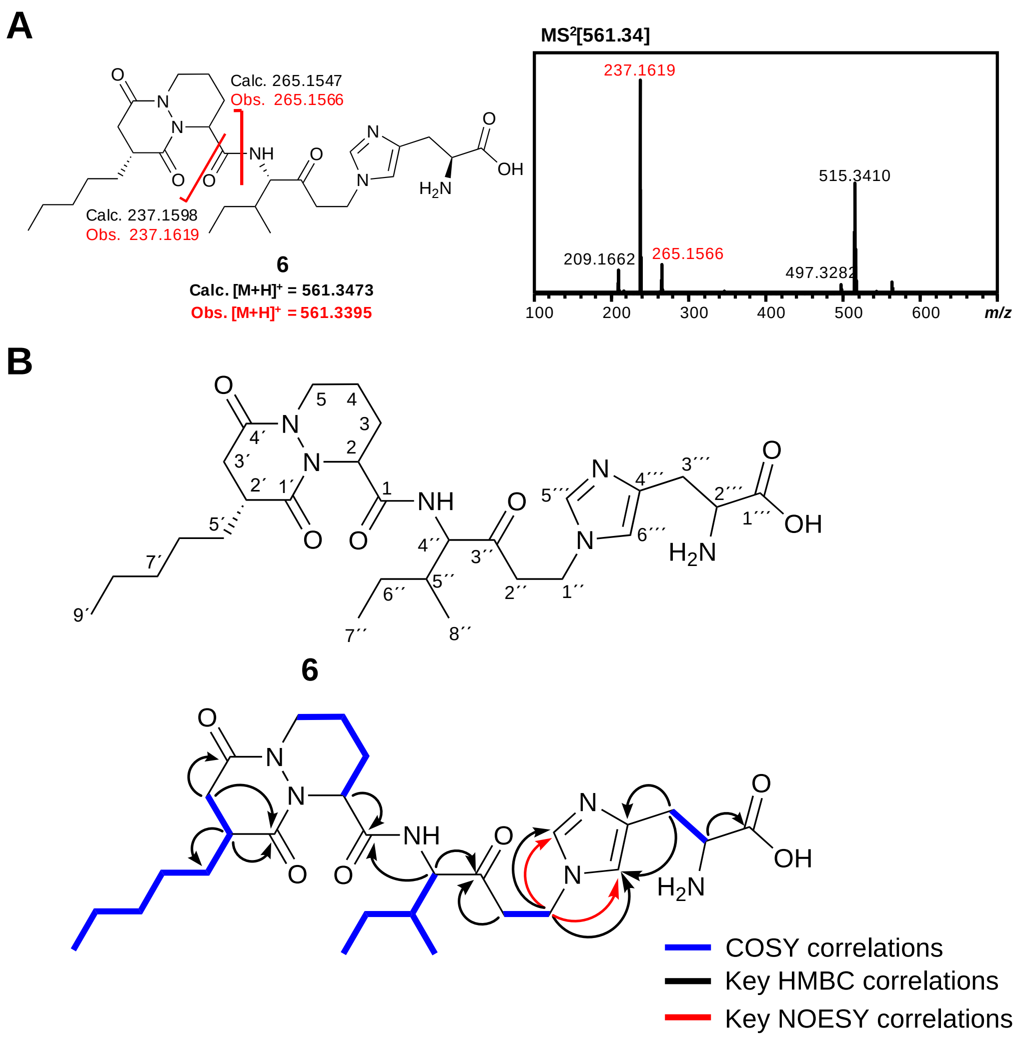

Supplementary Figure 24. Characterization of His-matlystatin derivative **6**. (A) MS^2 data for **6** acquired via method 2. (B) Atom numbering for **6** and key 2D NMR correlations identified for this compound. Complete NMR assignments are provided in Table S7 and original NMR data are shown in Figures S25-S30. MS data were acquired via method 2.

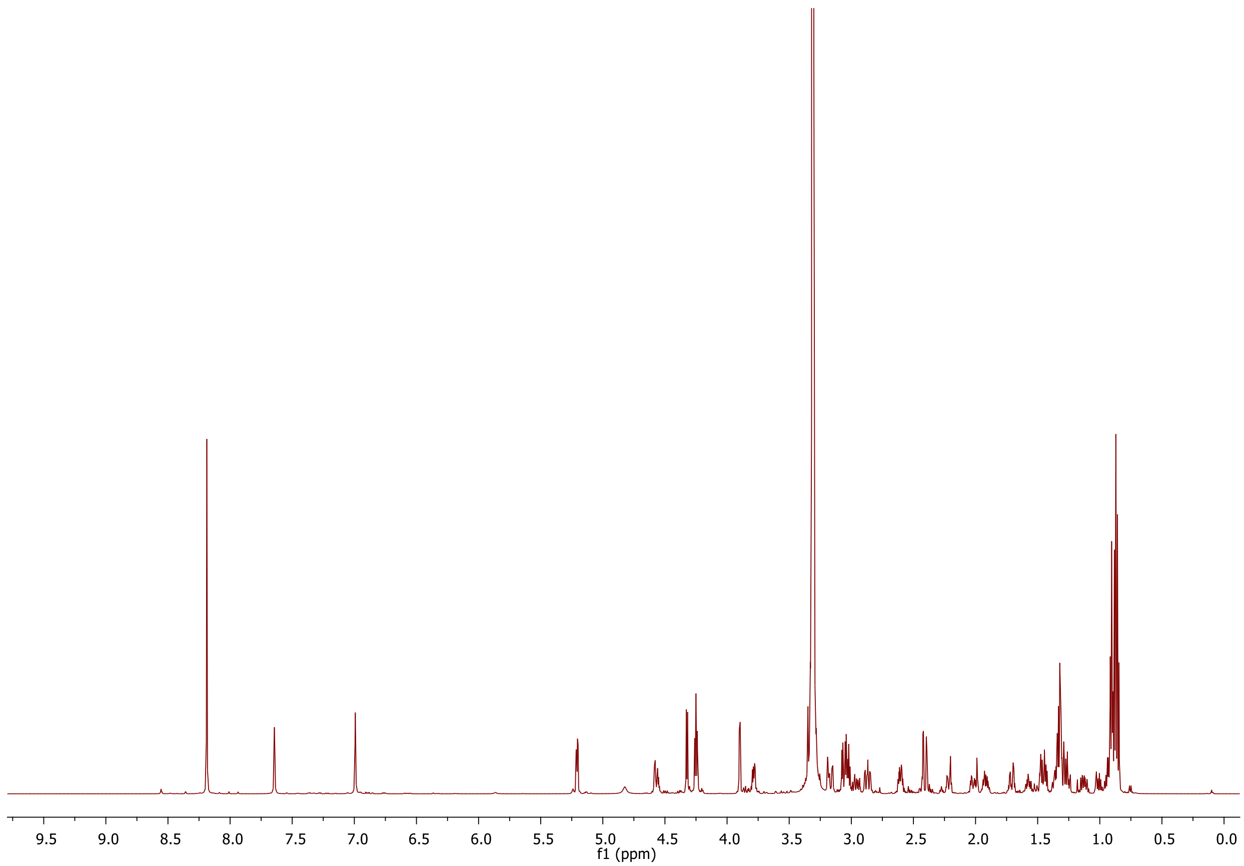

Supplementary Figure 25. 600 MHz ^{1}H NMR spectrum of **6** in CD_3OD.

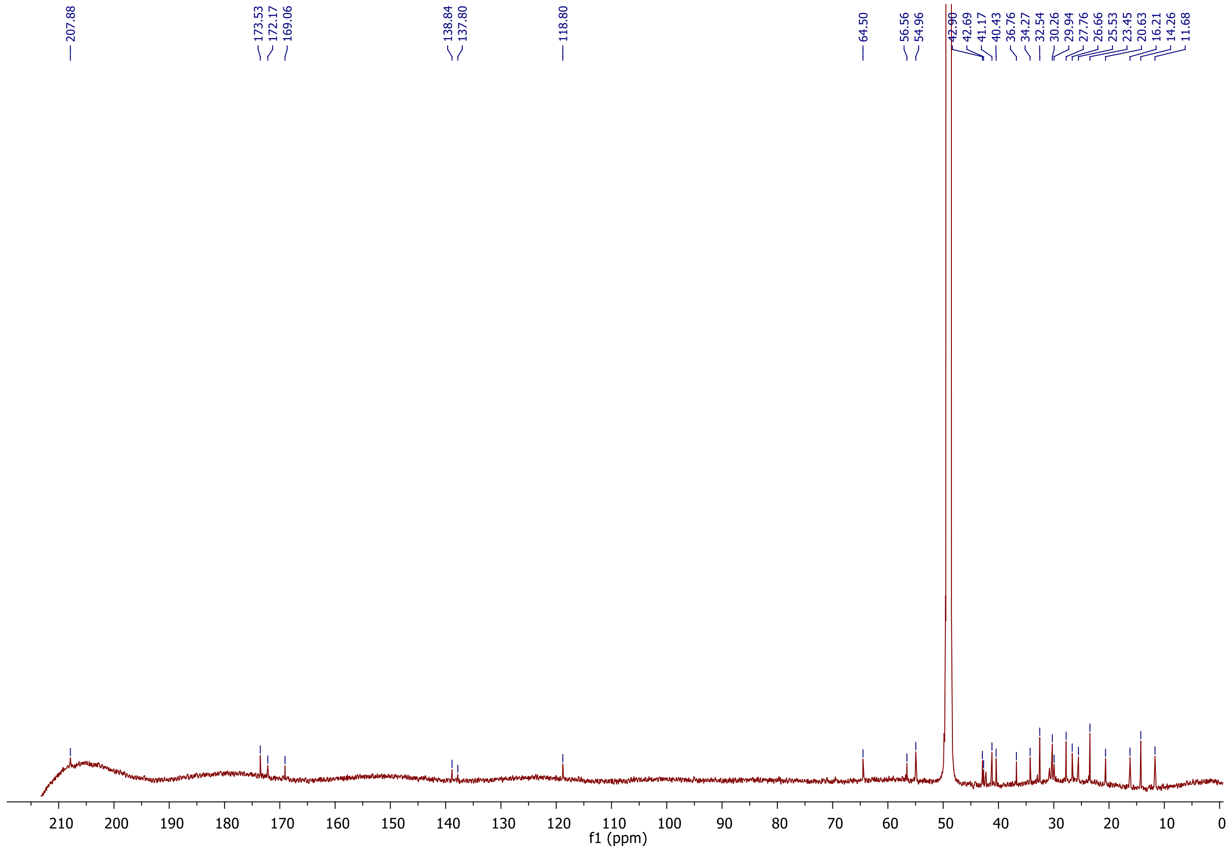

Supplementary Figure 26. 150 MHz ^{13}C NMR spectrum of **6** in CD_3OD.

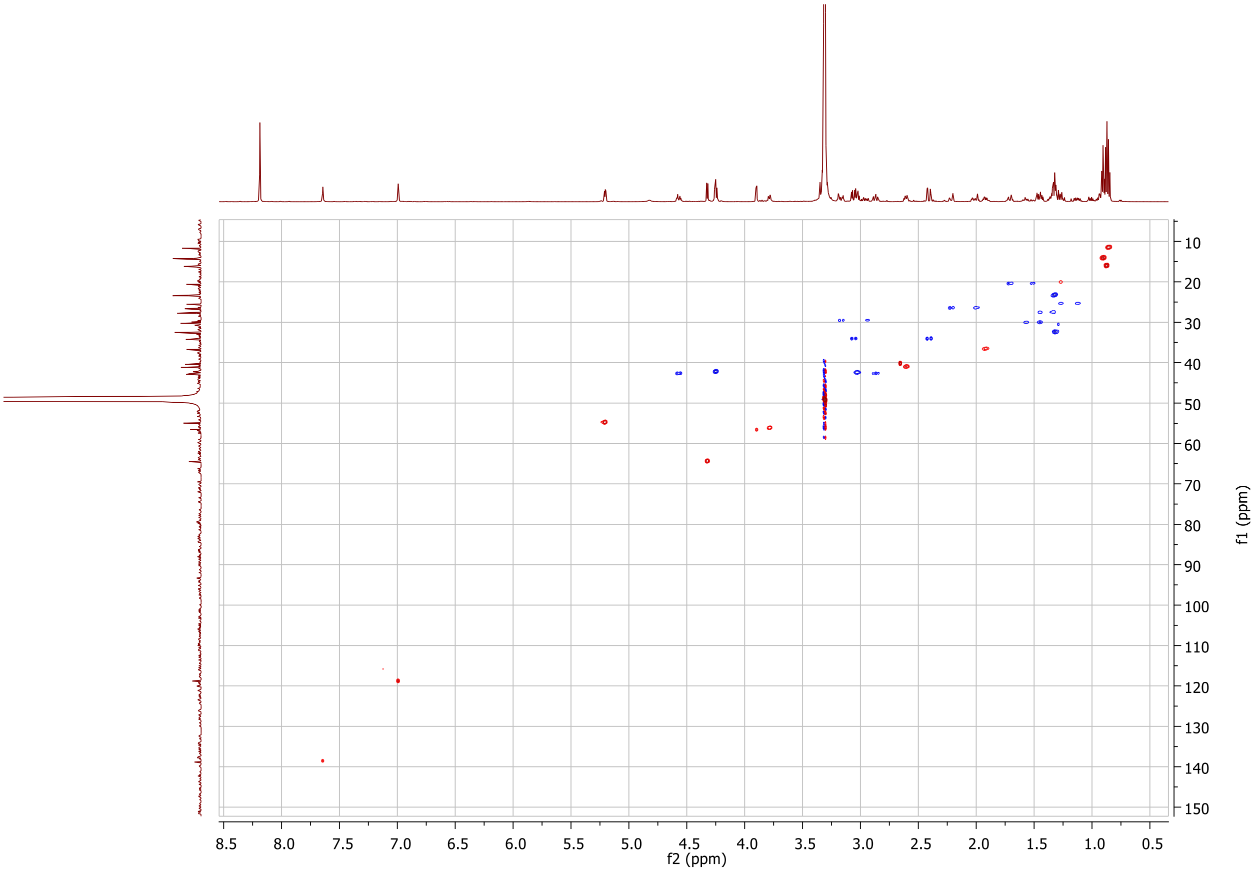

Supplementary Figure 27. 600 MHz ^{1}H HSQC NMR spectrum of **6** in CD_3OD.

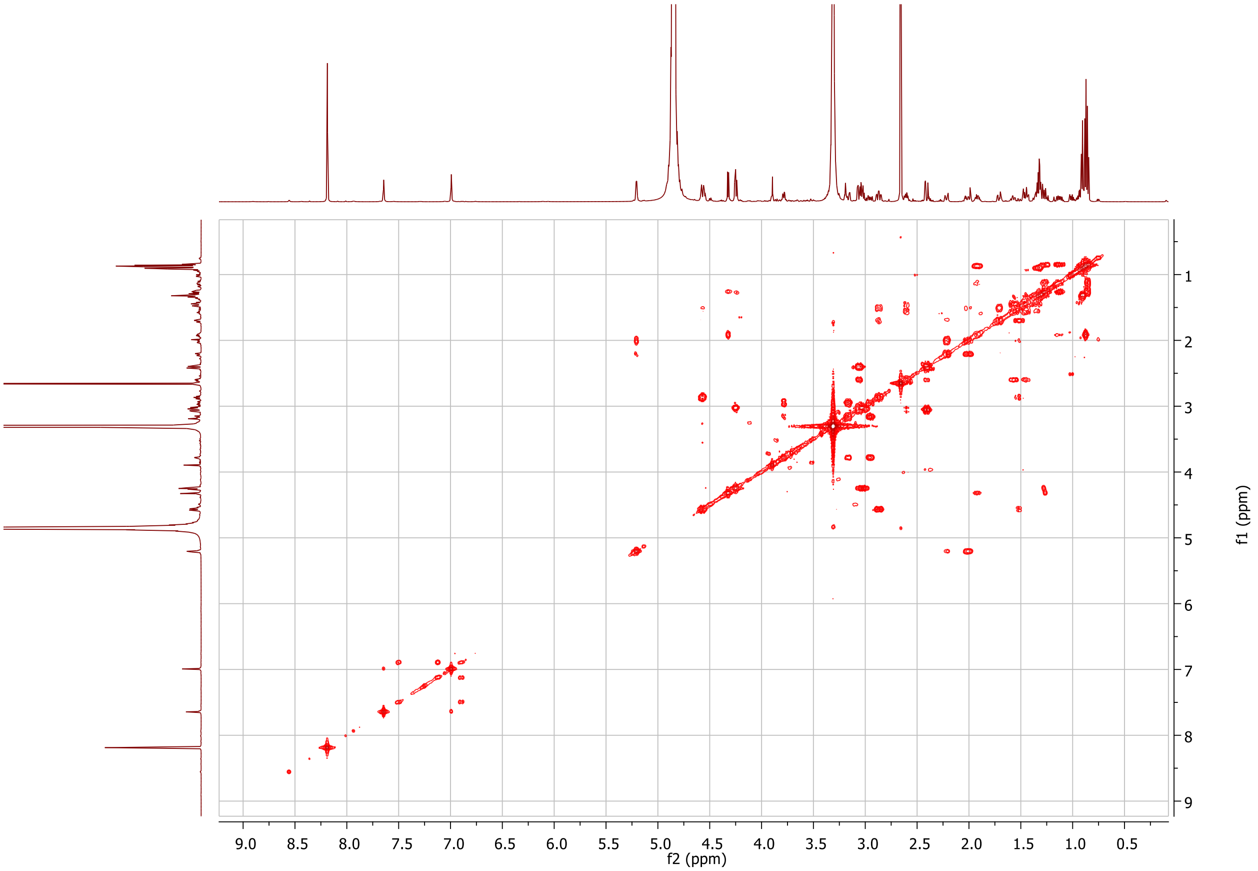

Supplementary Figure 28. 600 MHz ^{1}H-^{1}H COSY NMR spectrum of **6** in CD_3OD.

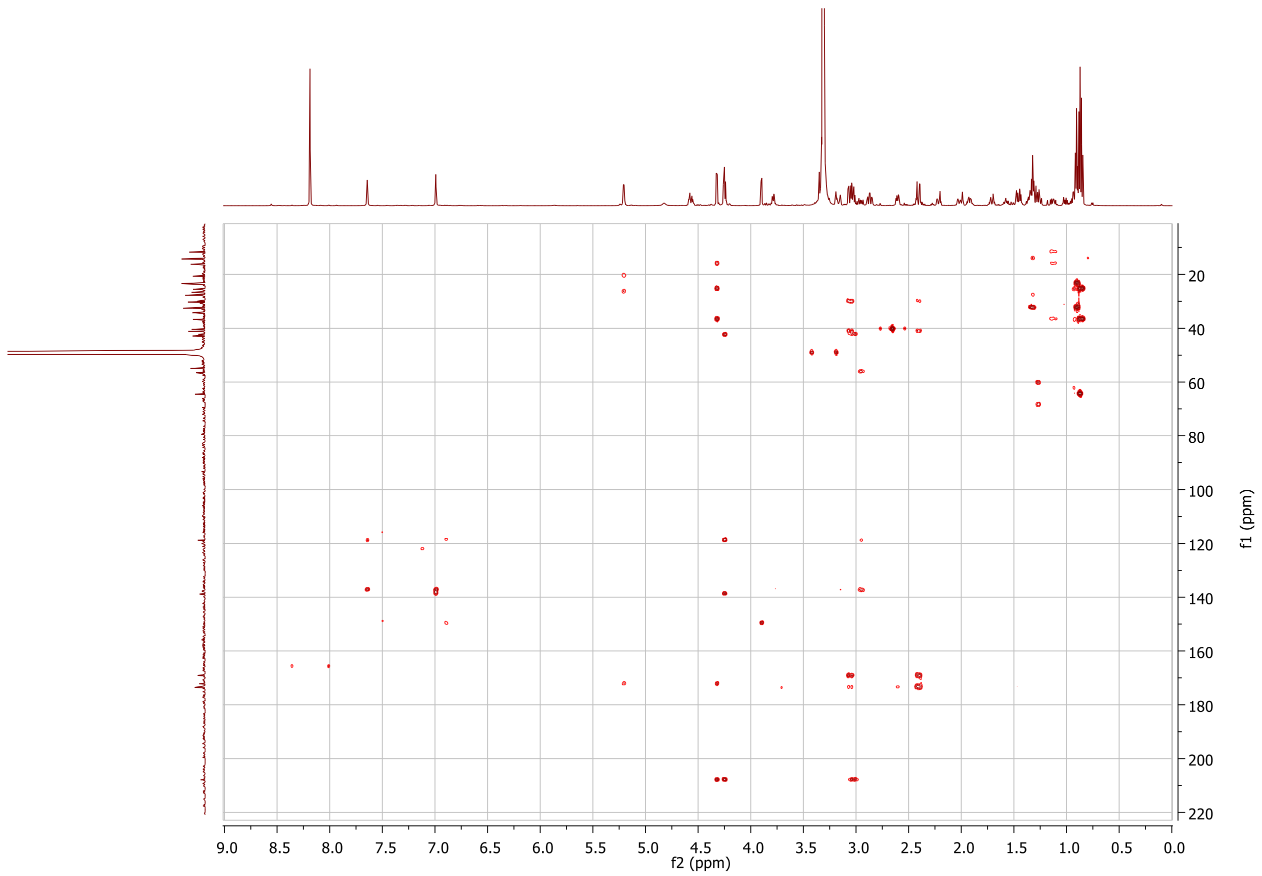

Supplementary Figure 29. 600 MHz ^{1}H-^{13}C HMBC NMR spectrum of **6** in CD_3OD.

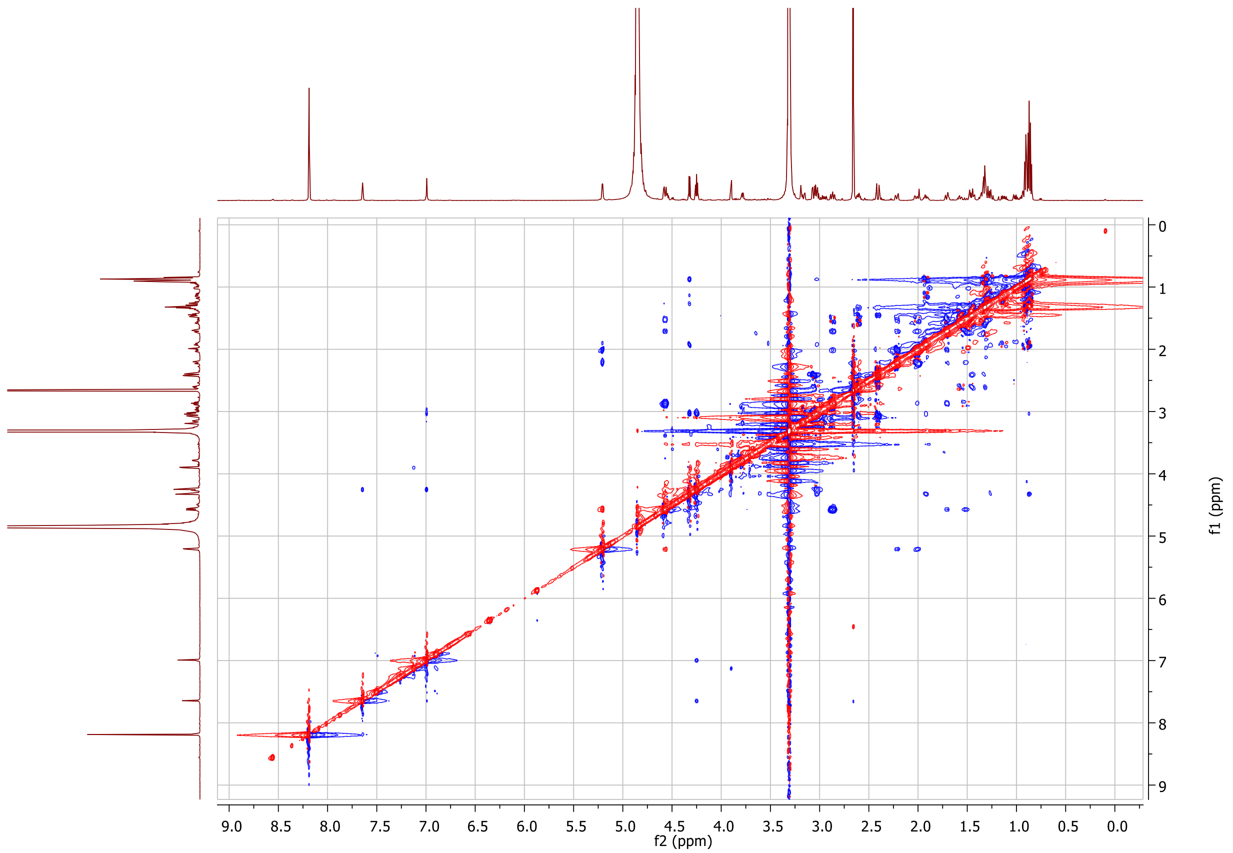

Supplementary Figure 30. 600 MHz ^{1}H-^{1}H NOESY NMR spectrum of **6** in CD_3OD.

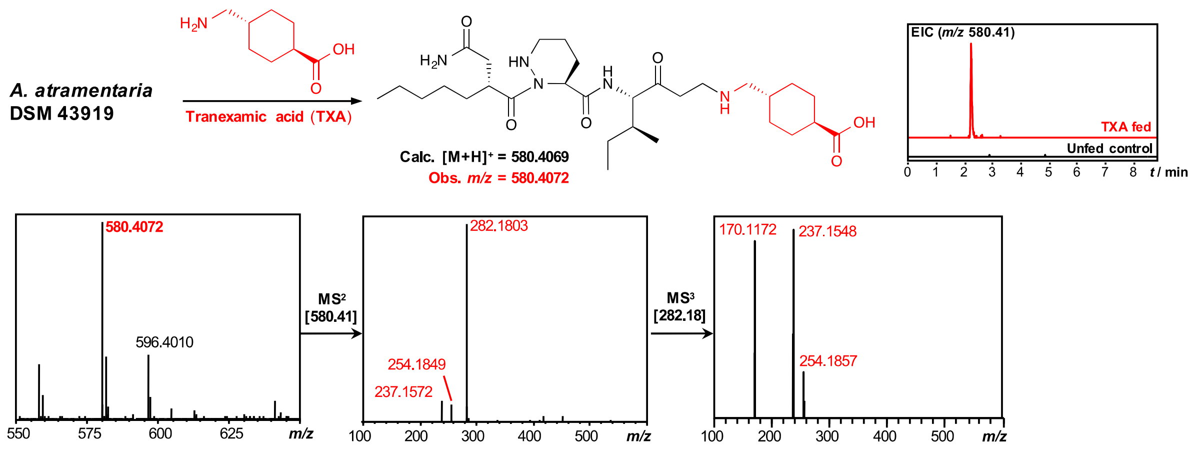

Supplementary Figure 31. LC-MS^n analysis of the product of feeding tranexamic acid to *A. atramentaria*. MS^2 and MS^3 fragments are highlighted that are consistent with the fragmentation of known matlystatin congeners. Data were acquired via method 2.

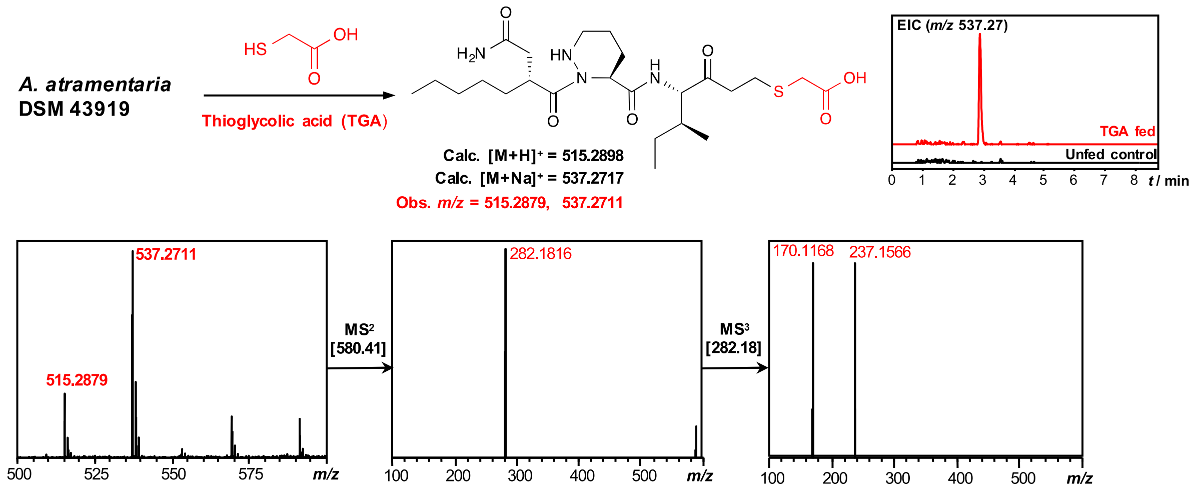

Supplementary Figure 32. LC-MS^n analysis of the product of feeding thioglycolic acid to *A. atramentaria*. MS^2 and MS^3 fragments are highlighted that are consistent with the fragmentation of known matlystatin congeners. Data were acquired via method 2.

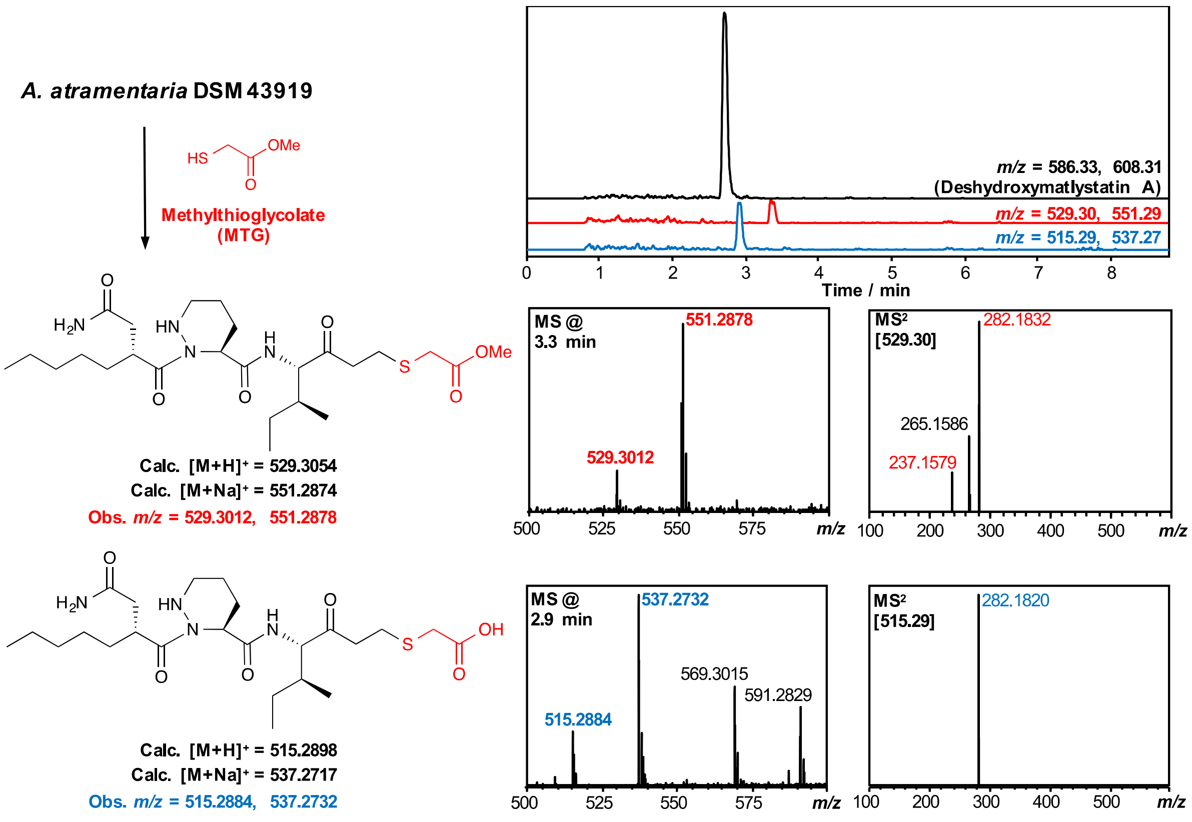

Supplementary Figure 33. LC-MS^n analysis of the product of feeding methylthioglycolate to *A. atramentaria*. MS^2 fragments are highlighted that are consistent with the fragmentation of known matlystatin congeners. The production of the thioglycolic acid adduct is consistent with hydrolysis of the methyl ester. Data were acquired via method 2.

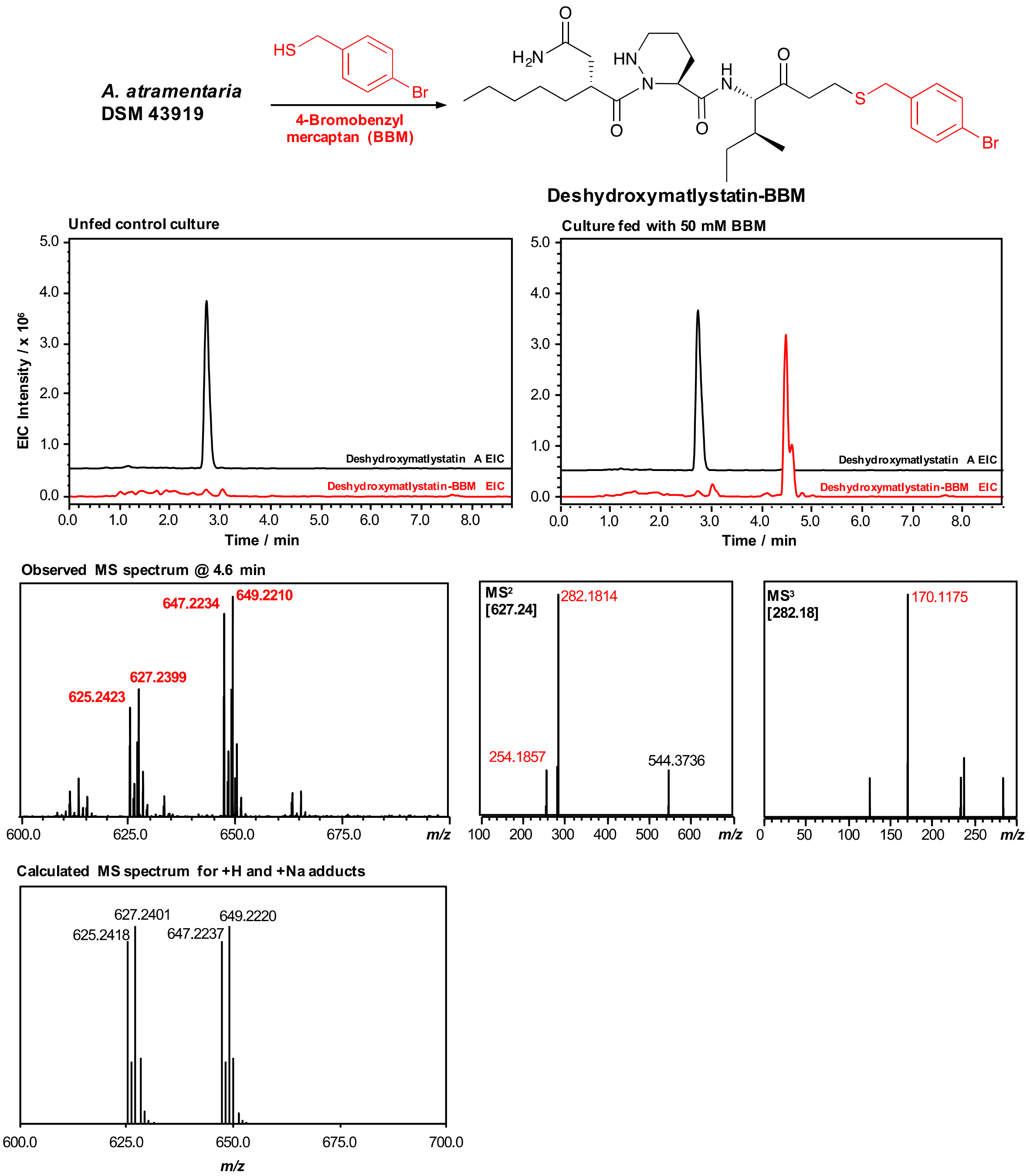

Supplementary Figure 34. LC-MS^n analysis of the product of feeding 4-bromobenzyl mercaptan (BBM) to *A. atramentaria*. MS^n fragments are highlighted that are consistent with the fragmentation of known matlystatin congeners. The calculated MS spectrum for proton and sodium adducts was generated using enviPat (http://www.envipat.eawag.ch/index.php). Data were acquired via method 2.

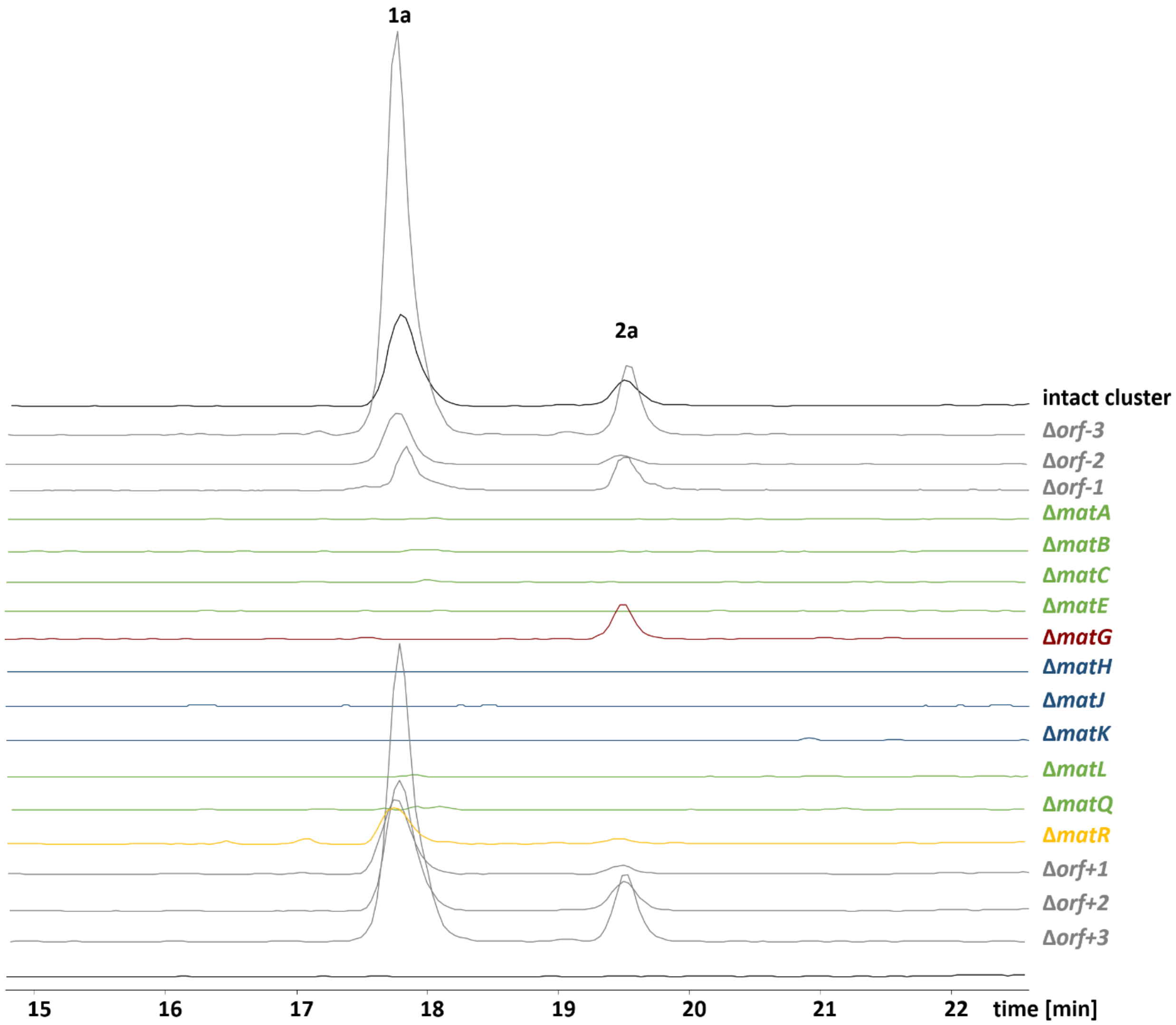

Supplementary Figure 35. LC-MS analysis of *mat* mutant strains. Extracted ion chromatograms (EICs) from culture extracts of *S. coelicolor* M1154 derivatives for deshydroxymatlystatin A (**1a**) *m/z* 586.3 $[M+H]^+$ and deshydroxymatlystatin B (**2a**) *m/z* 425.3 $[M+H]^+$. The heterologous host without and with the intact *mat* gene cluster is compared to strains with a mutant version of the cluster containing in frame *mat* gene deletions. Data were acquired via method 1.

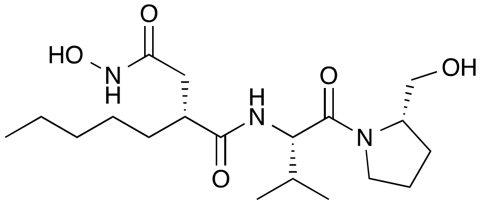

Actinonin

Unlabelled: Calc. [M+H]+ = 386.2649
$^{13}C_4$-labelled: Calc. [M+H]+ = 390.2784

Unlabelled: Calc. [M+Na]+ = 408.2469
$^{13}C_4$-labelled: Calc. [M+Na]+ = 412.2603

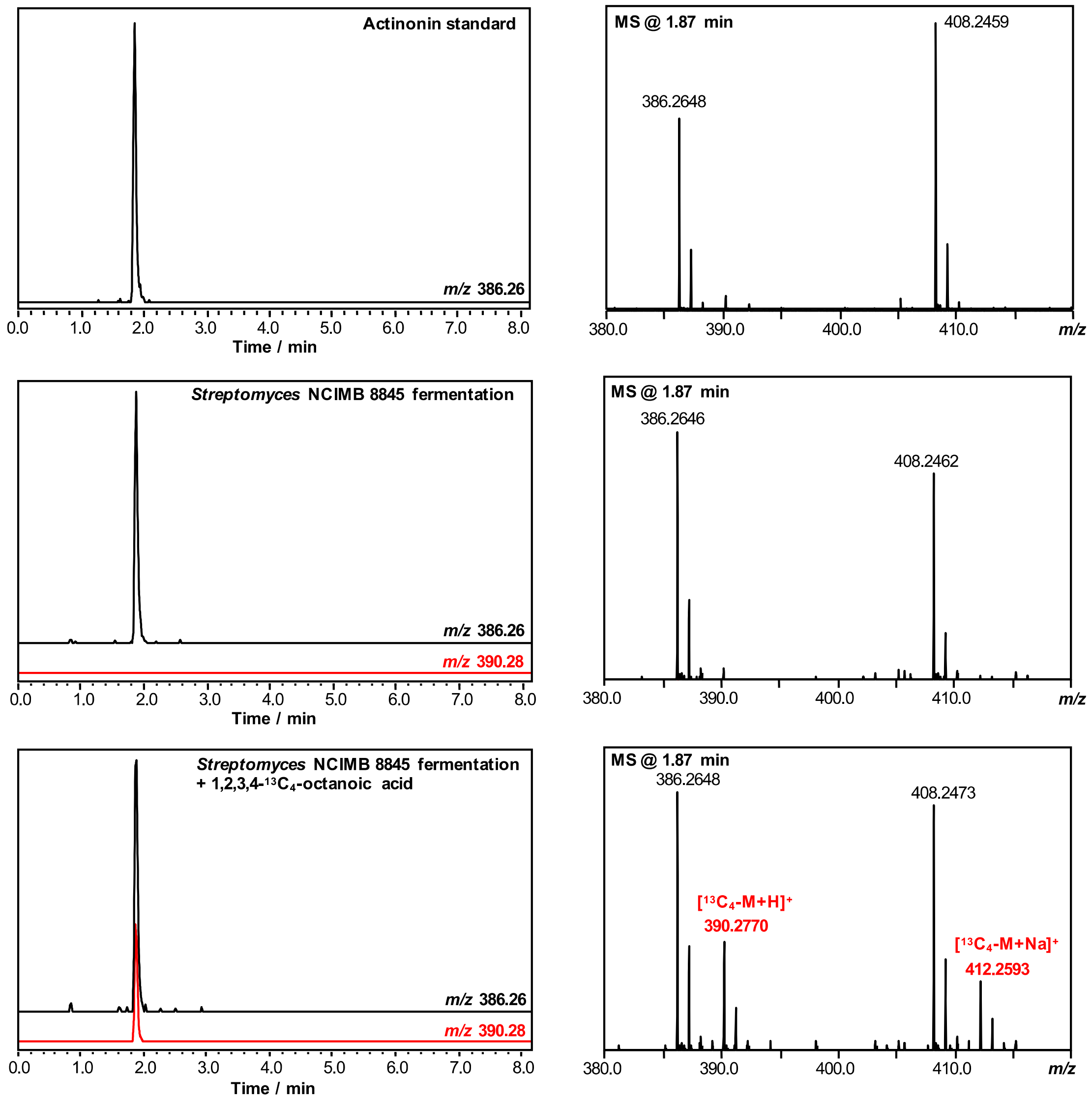

Supplementary Figure 36. Comparison of actinonin standard, actinonin produced by *Streptomyces* NCIMB 8845 and actinonin produced by *Streptomyces* NCIMB 8845 fed with [1,2,3,4-$^{13}C_4$]octanoic acid. LC-MS traces shown with positive mode MS data. Data were acquired via method 2.

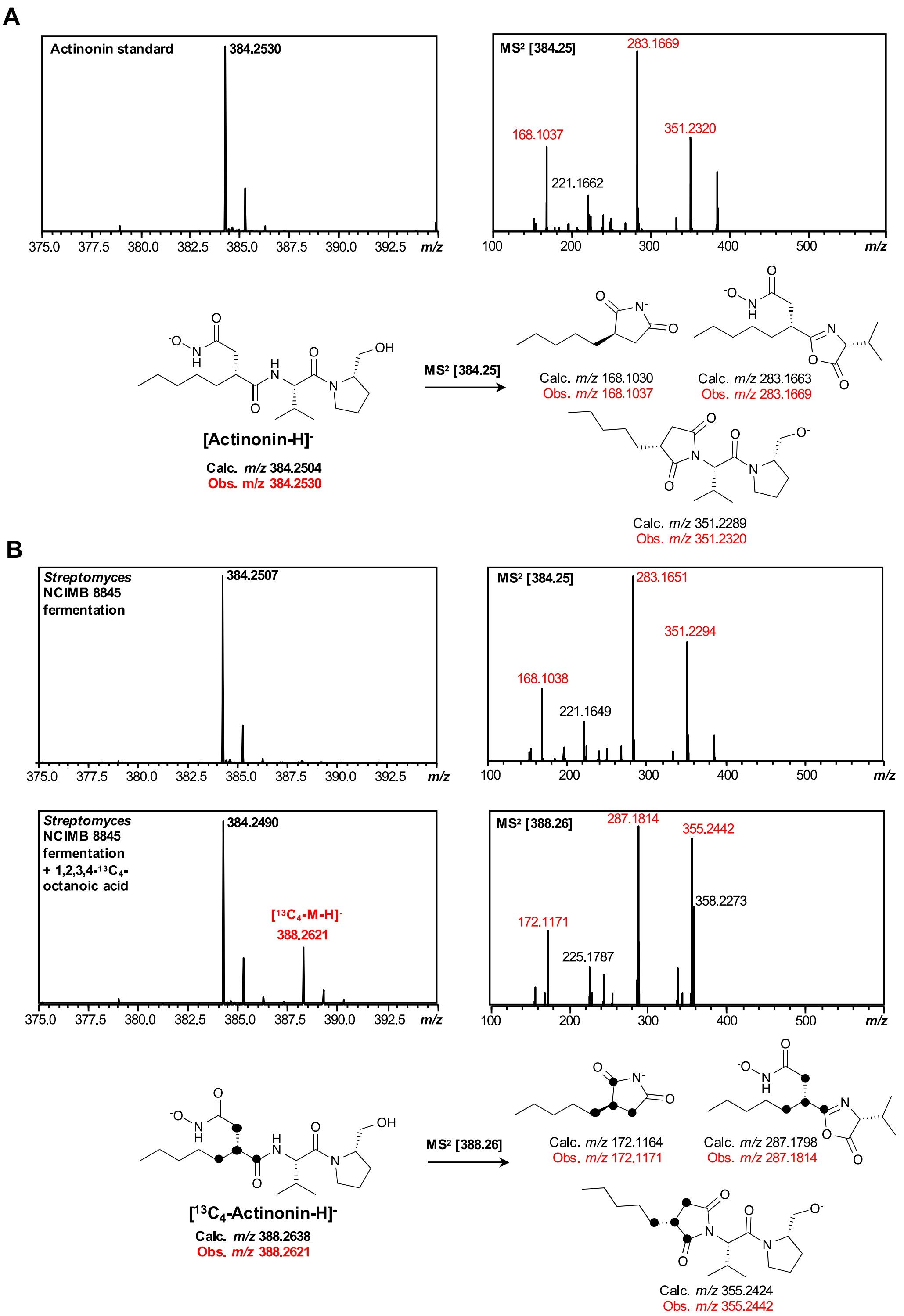

Supplementary Figure 37. Negative mode LC-MS^2 analysis of actinonin and $^{13}C_4$-actinonin. (A) Spectra and predicted fragmentation products for actinonin standard. (B) Spectra and predicted fragmentation products for actinonin and $^{13}C_4$-actinonin produced by *Streptomyces* NCIMB 8845. Data were acquired via method 2.

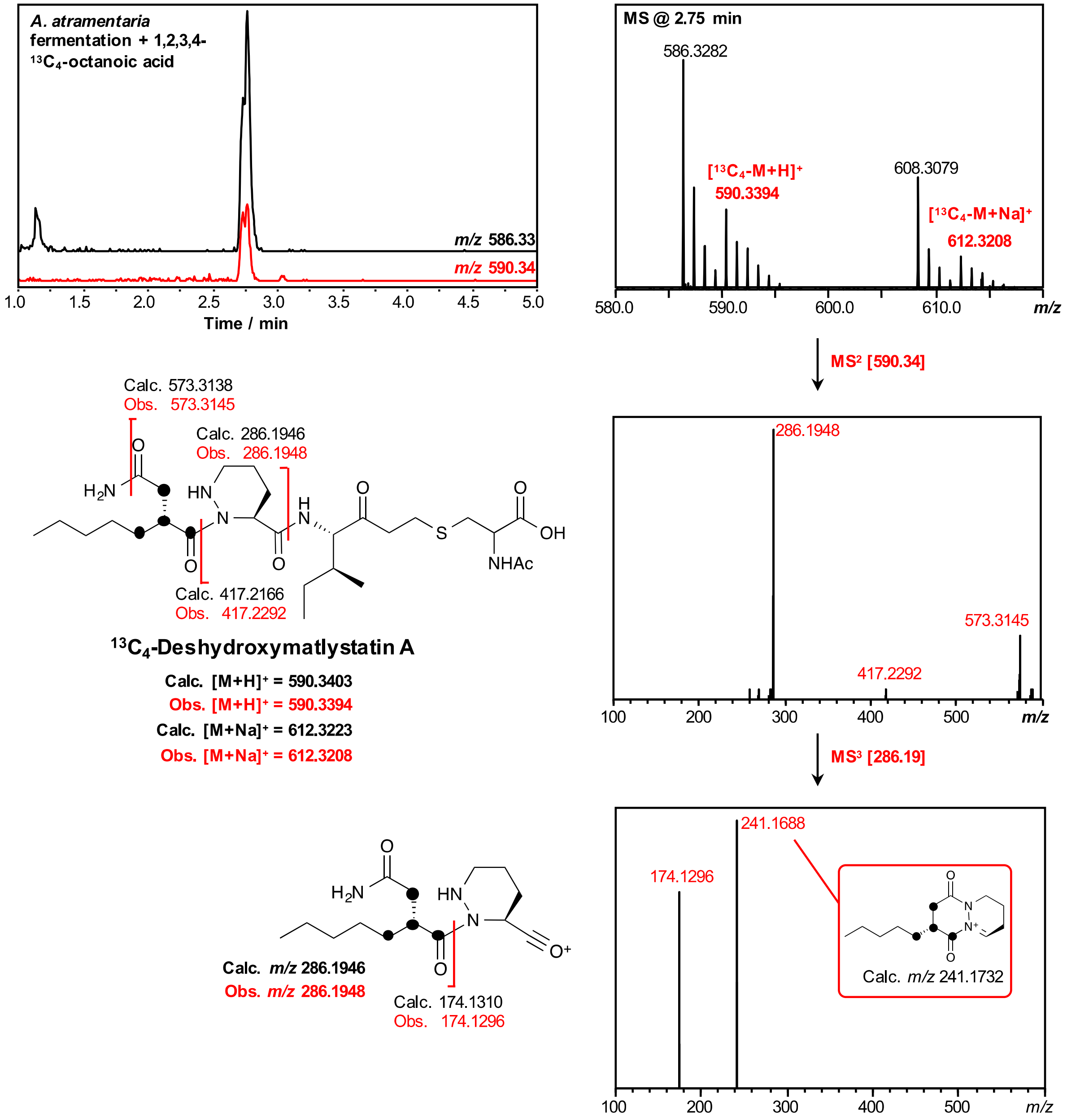

Supplementary Figure 38. LC-MS^n analysis of $^{13}C_4$-deshydroxymatlystatin A produced by *A. atramentaria* fed with [1,2,3,4-$^{13}C_4$]octanoic acid. Data were acquired via method 2.

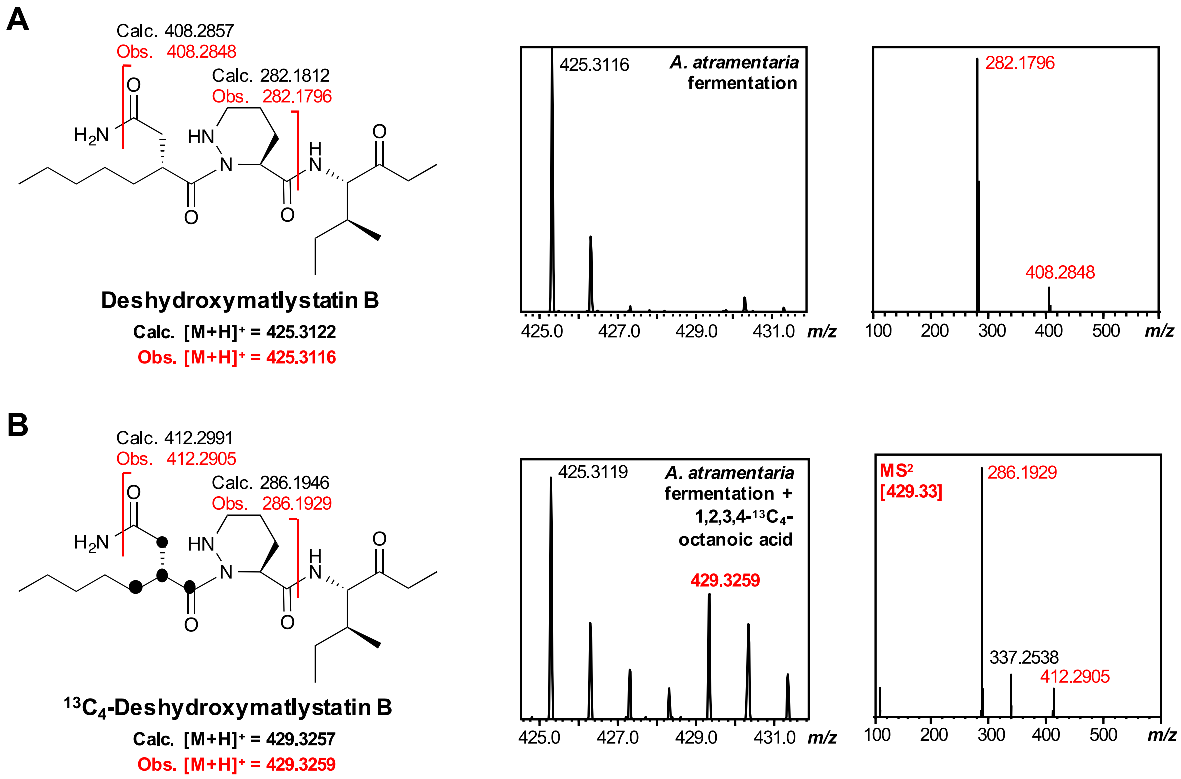

Supplementary Figure 39. LC-MS^2 analysis of deshydroxymatlystatin B and $^{13}C_4$-deshydroxymatlystatin B produced by *A. atramentaria*. (A) MS and MS^2 spectra for a control fermentation fed with unlabeled octanoic acid. (B) MS and MS^2 spectra for *A. atramentaria* fed with [1,2,3,4-$^{13}C_4$]octanoic acid. Data were acquired via method 2.

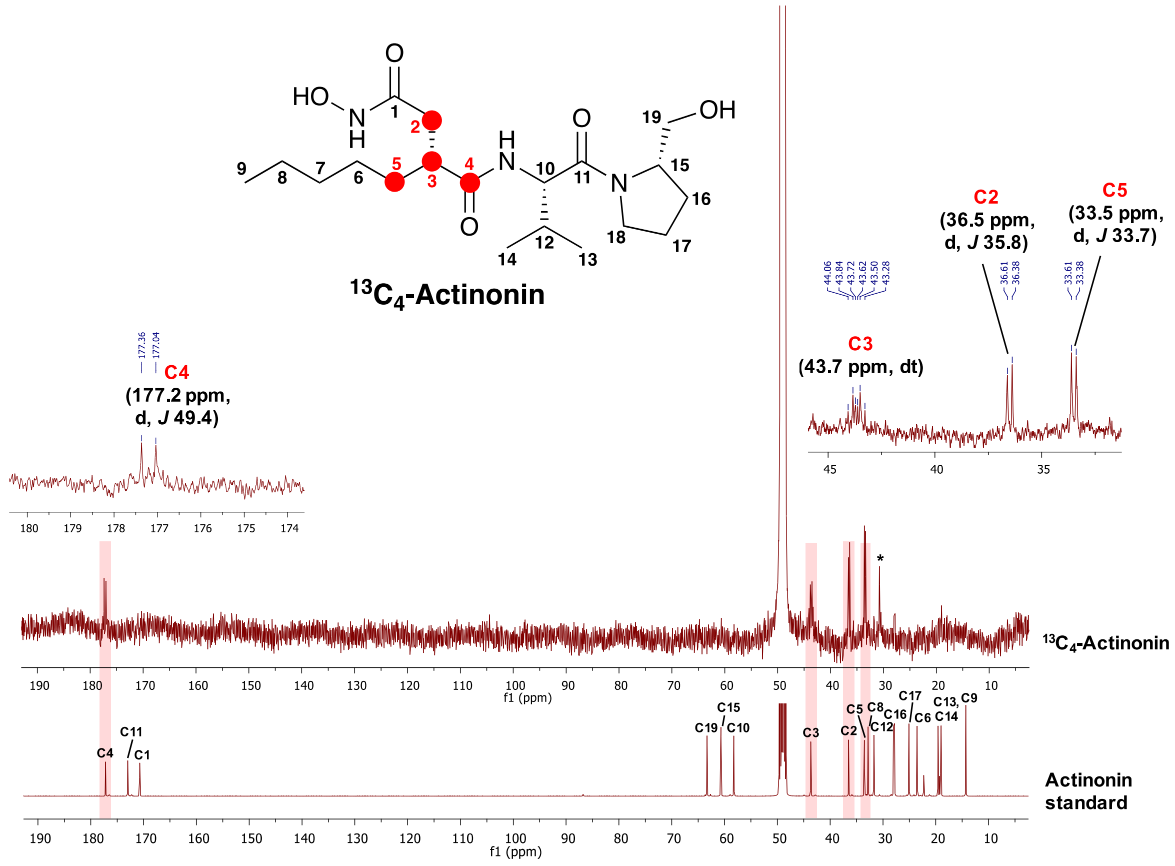

Supplementary Figure 40. 150 MHz ^{13}C NMR analysis of $^{13}C_4$-actinonin showing shifts and splitting patterns for carbons 2, 3, 4 and 5. The low amount of material prevented a full ^{13}C NMR analysis, so the ^{13}C NMR spectrum of an actinonin standard is shown for reference. The doublet of triplets at C3 arises from the similar coupling constants of C3 with both C2 and C5. The starred peak is a minor contaminant.

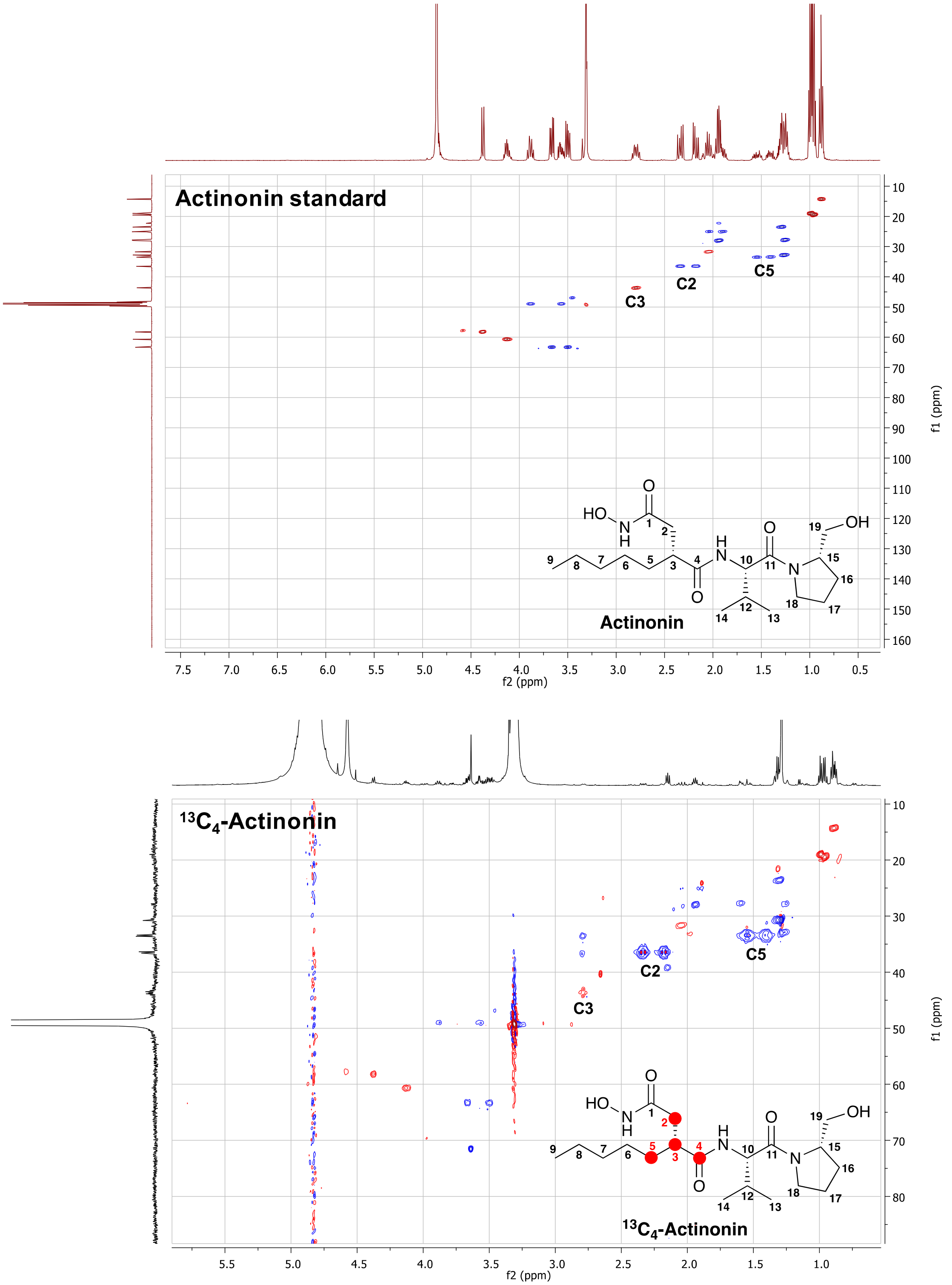

Supplementary Figure 41. A comparison of 600 MHz HSQC NMR data for $^{13}C_4$-actinonin and an actinonin standard confirming the identity of C2, C3 and C5 in $^{13}C_4$-actinonin.

Supplementary Table 1. Deduced functions of open reading frames in the matlystatin BGC

Gene	AA	Protein homolog	Accession number	identity / similarity[a]	Predicted function
orf-3	753	SAMN04489726_1294, *Allokutzneria albata*	SDM37208	58 / 64	cytochrome P450 + terpene synthase domain
orf-2	206	SAMN05421811_12520, *Nonomuraea wenchangensis*	SEU45505	75 / 85	TetR-like transcriptional regulator
orf-1	305	Tcur_0722, *Thermomonospora curvata*	ACY96315	73 / 83	alcohol dehydrogenase
matA	295	TUE45_03041, *S. reticuli*	CUW28310	45 / 60	N-oxygenase
matB	481	GA0115244_12547, *Streptomyces* sp. DvalAA-19	SCE40150	77 / 85	mutase
matC	618	BE04_36800, *Sorangium cellulosum*	KYF49978	53 / 67	asparagine synthase
matD	432	SAMN05421805_10330, *Saccharopolyspora antimicrobica*	SFN16255	59 / 72	L-ornithine N5-oxygenase
matE	139	SF12_13795, *S.* sp. MBRL 601	KIX77439	68 / 78	methylmalonyl-CoA epimerase
matF	232	GA0115234_10849, *S.* sp. DvalAA-43	SCE42322	53 / 65	transcriptional regulator
matG	546	EpxF, *Goodfellowiella coeruleoviolacea*	AHB38499	43 / 59	acyl-CoA dehydrogenase
matH	420	SAMN05443572_103577, *Myxococcus fulvus*	SET86746	37 / 51	non-ribosomal peptide synthetase C-domain
matI	83	GA0115244_12548, *Streptomyces* sp. DvalAA-19	SCE40160	56 / 72	phosphopantetheine attachment site
matJ	521	ABE83_07175, *Streptomyces* sp. CFMR 7	ALC31766	65 / 75	non-ribosomal peptide synthetase A-domain
matK	604	GA0115244_125410, *Streptomyces* sp. DvalAA-19	SCE40181	53 / 62	non-ribosomal peptide synthetase C-A-domain
matL	447	GA0115244_125411, *Streptomyces* sp. DvalAA-19	SCE40188	75 / 84	crotonyl-CoA reductase
matM	199	GA0070558_10148, *Micromonospora haikouensis*	SCE63615	60 / 67	transcriptional regulator
matN	520	A2W26_08340, *Acidobacteria bacterium* RBG_16_64_8	OFV81068	36 / 58	MFS transporter
matO	1548	DB31_0222, *Hyalangium minutum*	KFE71961	43 / 58	non-ribosomal peptide synthetase + polyketide synthase
matP	311	EsmB5, *S. antibioticus*	AFB35636	50 / 63	TE-domain, MbtH domain
matQ	135	GA0115244_125412, *Streptomyces* sp. DvalAA-19	SCE40202	73 / 84	methylmalonyl-CoA mutase
matR	403	SAMN04489717_5495, *Actinopolymorpha singaporensis*	SDT20828	62 / 76	LAO/AO transport system ATPase
orf+1	231	SAMN04489712_11710, *Actinomadura echinospora*	SEG84476	88 / 92	TetR family transcriptional regulator
orf+2	223	SAMN04489712_11711, *A. echinospora*	SEG84482	88 / 94	3-ketoacyl-ACP reductase
orf+3	68	BN4615_P5655, *Nonomuraea* sp. ATCC 39727	SBO96139	93 / 98	4-oxalocrotonate tautomerase

[a] amino acid sequence homology [%] from blastp analysis.

Supplementary Table 2. Deduced functions of open reading frames in the actinonin BGC

Gene	AA	Protein homolog	Accession number	identity / similarity[a]	Predicted function
orf-3	348	SAMN05421805_101959, *Sacch. antimicrobica*	SFM62800	53 / 65	LacI family TR
orf-2	428	Gobs_1988, *G.obscurus* DSM 43160	ADB74682	46 / 63	MFS transporter
orf-1	116	SAMN05421805_101958, *Sacch. antimicrobica*	SFM62777	60 / 71	beta-glucosidase
actA	677	B1H18_00560, *S. tsukubensis*	OON82982	64 / 73	dipeptidyl-peptidase
actB	498	GA0115243_101896, *Streptomyces* sp. ScaeMP-e83	SCD42850	68 / 84	ABC transporter
actC	151	MatE, *A. atramentaria* DSM 43919	WP_026341874.1	67 / 75	methylmalonyl-CoA epimerase
actD	481	MatB, *A. atramentaria* DSM 43919	WP_019634562.1	70 / 80	methylmalonyl-CoA mutase
actE	1596	MatO, *A. atramentaria* DSM 43919	WP_019634550.1	40 / 50	NRPS
actF	438	MatL, *A. atramentaria* DSM 43919	WP_019634553.1	67/78	crotonyl-CoA reductase
actG	822	MatK, *A. atramentaria* DSM 43919	WP_026341873.1	38 / 50	NRPS
actH	474	MatO, *A. atramentaria* DSM 43919	WP_019634550.1	36 / 46	adenylation domain
actI	316	MatA, *A. atramentaria* DSM 43919	WP_019634563.1	48 / 64	AurF-like N-oxygenase
actJ	618	MatC, *A. atramentaria* DSM 43919	WP_019634561.1	58 / 71	asparagine synthetase B
actK	462	AXA44_32810, *Rhodococcus* sp. SC4	KXF56755	42 / 57	amidase
actL	224	Y710_16995, *Gordonia* sp. QH-12	KXT55822	34 / 53	TetR family TR
orf+1	522	QF38_RS40510, *Mesorhizobium* sp. F7	WP_063770482.1	42 / 62	MFS transporter
orf+2	679	SM007_23995, *S. avermitilis*	OOV26101	83 / 89	short-chain dehydrogenase
orf+3	177	SAMN02787144_104422, *S. atratus*	SFY44206	77 / 85	membrane protein

[a] amino acid sequence homology [%] from blastp analysis

*Abbreviations

AA	protein length in amino acids
BGC	biosynthetic gene cluster
TR	transcriptional regulator
NRPS	non-ribosomal peptide synthetase
S.	*Streptomyces*
A..	*Actinomadura*
Sacch.	*Saccharopolyspora*
G.	*Geodermatophilus*

Supplementary Table 3. Analysis of A- and AT-domains in the matlystatin and actinonin gene clusters: Predicted substrate specificities according to AntiSMASH analysis.

Protein	Domain	Predicted substrate [a]	Postulated function
MatJ	A1	nrp[b]	activation of piperazic acid
MatO	A2	leu	activation of isoleucine
	AT	mal	activation of methylmalonate
ActE	A1	pro	activation of proline
ActG	A2	-	unknown
ActH	A3	thr	activation of valine

a] Consensus of substrate specificities predicted by used algorithms: NRPSPredictor2 SVM, Stachelhaus code, Minowa for A-domains and PKS signature, Minowa for AT-domains. [b] nrp: undefined/no consensus

Supplementary Table 4. Primers used in this study

Primer name	Primer sequence (5'-3')
mat_f	CTGGTCATGAAGAGACTCGC
mat_r	CAGCGACGTGATGTCCTTCG.
orf-3_F	CGCGCCGCGTGCGCACCGGGCGGCCCGGCCCGGCCGTCA**TGTAGGCTGGAGCTGCTTC**
orf-3_R	GACCGGGAACGACCCGGTCACCCGGGACGACGGCCCGTG**ATTCCGGGGATCCGTCGACC**
orf-3_test_F	GCCAGCACGACCGAAATGAC
orf-3_test_F2	GAGGAGCGGTCGGGCCATCTGC
orf-3_test_R	TTGCGAAGCATGCCGACCGTTG
orf-2_F	CGGTCCCGGACGGGCCGTGCGGGTTTCGGTCGTCCGTTA**TGTAGGCTGGAGCTGCTTC**
orf-2_R	CGGACCGGCAGTCCGATTGAGGAGCGTGACCCCGCGATG**ATTCCGGGGATCCGTCGACC**
orf-2_test_F	GCGGGAAGTTACCATATTCG
orf-2_test_R	GACTTGACCCTTGCTTTACC
orf-1_F	TGAATGCCGGGCGCCCCGTTACGCGGCGGCGGGTTCGAC**ATTCCGGGGATCCGTCGACC**
orf-1_R_alt	TTGGAGCGCTCCATGCCCATGCAAGCCCTCGTCGTCGAC**TGTAGGCTGGAGCTGCTTC**
orf-1_test_F	GCCGGTCCGATTCAGAGAAG
orf-1_test_R	GGACTGCCGGTCCGAATATG
matA_f	CCCGCCTCGTACGGGAACTCGCCTGGGCCCGCCATCTCA**ATTCCGGGGATCCGTCGACC**
matA_r	AGTGCTGTTTTCTCGCGTTGGCACGAACGTGCGGGGGTG **TGTAGGCTGGAGCTGCTTC**
matA_test_F	GTGTAACCGGTCGGATGAATGC
matA_test_F2	ACCTCCAGGGCCCGGTTCGCGC
matA_test_R	CTCCGAGCGATTTGGGTCATTG
matB_f	TTGTCTCCTCGTTTCGCTTGGTTGCCGGTCGGCCGGTCA**ATTCCGGGGATCCGTCGACC**
matB_r	TCGAGGCGTCCGGGCTCCTGATCGGGGAGATCTGAGATG**TGTAGGCTGGAGCTGCTTC**
matB_test_F	ATGGTTGTCTCCTCGTTTCG
matB_test_F2	CCGGGGTCGACCTCGAACATCC
matB_test_R	GGGAGATCTGAGATGTGTAG
matC_f	CCAGGATGTCGTCGGCGTCGGTCGCGGGGCCGGTCACAG**ATTCCGGGGATCCGTCGACC**
matC_r	GGCCGACCGGCAACCAAGCGAAACGAGGAGACAACCATG**TGTAGGCTGGAGCTGCTTC**
matC_test_F	GACGATGTCCAGGATGTC
matC_test_F2	GGTCGTCTCGGCGGCGCTCTGC
matC_test_R	GCTTCACCGAGCACTTTC
matE_f	TACTGGGGGGGAACGAACACTGGTCCTCCGAACGCGTCA**ATTCCGGGGATCCGTCGACC**
matE_r	CCGGTCCATCGCCGGGGCCCGGGTCGGGGCGGTGTGATG**TGTAGGCTGGAGCTGCTTC**
matE_test_F	TCCGCACCGTTTCGAGTGTC
matE_test_F2	GTCGATCAGCGCCGACAGCTCG
matE_test_R	ATCTCCGCGCAACTGCTGTC
matG_f	CGTGGGACGCCGGTCCCACGGCTCCGGCGACGGCCATCA**ATTCCGGGGATCCGTCGACC**
matG_r	GCCCGGCCGCGCGGGAGGCCGGCGATGGCGCGCGGTAGC**TGTAGGCTGGAGCTGCTTC**
matG_test_F	GAACTCGCGGAAGAACAC
matG_test_R	GTCGACGGGATGTACAAG
matH_F	TGACGGTCGCGACGGTCGTGGCGGTCGTGTCGGTCACGG**ATTCCGGGGATCCGTCGACC**
matH_R	CCGCATCAGCGCCGCCTGGCTGGGACCGGTGACCTGATG**TGTAGGCTGGAGCTGCTTC**
matH_test_f	GCGGTGACGGTCGCGACGGTCG
matH_test_r	GCACCTGGCGCGACTCGCCGGG
matJ_F	CGAGCTGCGCGGCGTTCGCCGGGCGCGTCGCGGTCACCG**ATTCCGGGGATCCGTCGACC**

matJ_R	GGCCGTGGACGAGCTGCGGGCGGGGGTGTCCCGGTGAGC**TGTAGGCTGGAGCTGCTTC**
matJ_test_f	TGCGCGGCGTTCGCCGGGCGCG
matJ_test_r	CGAGCTGCGGGCGGGGGTGTCC
matK_F	CCGGCGACGACGGCCTCGGCGAGAGCGTCCATGCGATCA**ATTCCGGGGATCCGTCGACC**
matK_R	GACCTCCTCCGACCGCCGCGCCGGGCGGTCCCGGTGACC**TGTAGGCTGGAGCTGCTTC**
matK_test_f	CGGCGAGAGCGTCCATGCGATC
matK_test_r	CCTCCGACCGCCGCGCCGGGCG
matL_f	CTTGCAAAGCAGGGGATCAAAGCGGCCGCCGGGCGGTCA**ATTCCGGGGATCCGTCGACC**
matL_r	TCGCCGAGACCGCCGAACCCGAGGAGGACTGATCGCATG**TGTAGGCTGGAGCTGCTTC**
matL_test_F1	CATGTGCAGCTCCATAAG
matL_test_F2	CGGAGGTGGAAGGAGTACAG
matL_test_F3	GTCGGGCACTGCGTGCTCCTCG
matL_test_R	GTCATCGAGATCGCCGAGAC
matQ_f	GCCGGTCCCCCGCCCGCGCCCGGTCCAGCAGGCTCACTC**ATTCCGGGGATCCGTCGACC**
matQ_r	CGCCCTCGCGCCCGGAACCAAGGAGGCGGACCGGTGAGA**TGTAGGCTGGAGCTGCTTC**
matQ_test_F	GCCTTGTTGACGACGTAGAC
matQ_test_R	CGCCGACTTCCAGATCTTC
matR_F	CGGACGCCTGACCGGGCACGCGGGCGGTGGGTCGGCTCA**ATTCCGGGGATCCGTCGACC**
matR_R	CCGGGCGGAGCTGCGGCCCCTCGAGGTGGCCGAGTGAGC**TGTAGGCTGGAGCTGCTTC**
matR_test_f	ACCGCTCTCCTCCAACGATG
matR_test_r	ATCTTCACGCCCAGCAGCTC
orf+1_F	ACGCGTTCTGAGCCGACCCACCGCCCGCGTGCCCGGTCA**TGTAGGCTGGAGCTGCTTC**
orf+1_R	GTATCACCGCTAGCAATCGGCGATATCATCGATCTCATG**ATTCCGGGGATCCGTCGACC**
orf+1_test_f	TCGGCGATATCATCGATCTC
orf+1_test_r	TCACAGCAGTGGTCATTCTC
orf+1_test_r2	GGCTCGCCGCCGCGGCGCTC
orf+2_F	CGGGCGGTGCTGCGGCGGCCTGCCGGGACGCGGCCGTCA**ATTCCGGGGATCCGTCGACC**
orf+2_R	AGCGGCGGCTCCCGCGGAATAGGGAGGGCCGTCGCGGTG**TGTAGGCTGGAGCTGCTTC**
orf+2_test_f	GGCATGCTGGTCACCTTTCG
orf+2_test_r	CAAGAGGAGGCAGCTATGAG
orf+3_f	GCCCGTTCCCGACCATCCCCACGAAAGGTGACCAGCATG**TGTAGGCTGGAGCTGCTTC**
orf+3_r	TGCCGGGGCCGGGCGGCCGCCCGGCCCCGGGGCGGGTCA**ATTCCGGGGATCCGTCGACC**
orf+3_test_F	CACGCGAACGTGATTGTG
orf+3_test_R	AACGGCGGGATCATCTGAC

Bold letters represent 39-nucleotide homologous extensions for Red/ET-mediated recombination

Supplementary Table 5. Spectroscopic data for deshydroxymatlystatin A

	major conformer		minor conformer			
atom	δ_H[b]	δ_C[c]	δ_H[b]	δ_C[c]	^1H-^1H-COSY	^1H-^{13}C-HMBC
1		173.0, qC		170.4, qC		NH of Ile, 2, 3, 4′
2	5.29, m	50.5, CH		53.7, CH	3	1, 1′w
3	1.85, m<br>2.20, m	26.7, CH_2	1.98, m<br>2.42, m	25.9	2, 4	1
4	1.53, m<br>1.60, m	21.7, CH_2	1.52, m<br>1.71, m	20.2	3, 5	
5	2.89, m<br>2.97, m	47.1, CH_2	4.55, m	41.4, CH_2	4, NH	
NH	4.97, d (11.8)		4.97, d (11.8)		5	1′w
1′		177.0, qC		177.0, qC		NHw, 2w, 2′, 3′, 5′
2′	3.86, m	36.6, CH	3.86, m	36.6, CH	3′, 5′	1′, 4′
3′	2.25, m<br>2.52, m	38.0 CH_2	2.25, m<br>2.52, m	38.0 CH_2	2′	NH_2, 1′, 4′
4′		173.8 qC		173.8 qC		2′, 3′
5′	1.40, m<br>1.51, m	32.7, CH_2	1.40, m<br>1.51, m	32.7, CH_2	2′	1′
6′	1.28, m<br>1.39, m<br>1.3	26.85, CH_2	1.28, m<br>1.39, m	26.81, CH_2		
7′	1.24, m	32.2, CH_2	1.27, m	31.5, CH_2		
8′	1.28, m	22.6, CH_2	1.28, m	22.5, CH_2	9′	
9′	0.86, t (7.1)	13.9 CH_3	0.86, t (7.1)	13.8 CH_3	8′	
NH_2	6.65, br s		6.65, br s			3′
1′′	2.79, m	26.12, CH_2	2.79, m	26.08, CH_2		
2′′	2.81, m<br>2.91, m	40.7, CH_2	2.81, m<br>2.91, m	40.6, CH_2		3′′
3′′		208.1, qC		207.9 qC		2′′, 4′′
4′′	4.36, t (7.1)	63.3, CH	4.36, t (7.1)	63.5, CH	NH of Ile, 5′	1, NH, 3′′
5′′	2.00, m	35.67, CH	2.00, m	35.69, CH	4′′, 6′′, 8′′	NH
6′′	1.19, m<br>1.43, m	24.9, CH_2	1.19, m<br>1.43, m	24.8, CH_2	5′′, 7′′	
7′′	0.86, t (7.1)	11.2, CH_3	0.86, t (7.1)	11.2, CH_3	6′′	
8′′	0.92, d (6.9)	15.8, CH_3	0.92, d (6.9)	15.8, CH_3	5′′	
NH of Ile	8.25, d[d]		8.22, d[d]		4′′	1, 4′, 5′′
1′′′		171.3, qC		166.5, qC		3′′′
2′′′	4.56, m	53.0, CH	4.56, m	53.0, CH	NH, 3′′′	NH, C=O of COAc
3′′′	2.88, m<br>3.04, m	34.2, CH_2	2.35, m<br>3.08, m	33.6, CH_2	2′′′	1′′′
NH	8.14, d (7.1)		8.12, d[d]		2′′′	2′′′, C=O of COAc
C=O of COAc		169.8, qC		172.8, qC		Ac, NH, 2′′′
Ac of COAc	1.97, s	22.3, CH_3	1.96, s	22.3, CH_3		C=O of COAc

[a] Measured at 400 (^1H) and 101 MHz (^{13}C) in d_7-*N*,*N*-DMF. [b] Coupling constants (*J*) are in parentheses and reported in Hz; s: singlet, d: doublet, m: multiplet, br: broad, w: weak; chemical shifts are given in ppm. [c] Multiplicities were deduced from an edited HSQC experiment. [d] Coupling was observed, but coupling constant could not be determined due to signal overlap resulting in turn from the presence of conformers.

Supplementary Table 6. Enrichment calculation for deshydroxymatlystatin A

carbon atom	δ_C [ppm] (major conformer)	integrals (^{13}C-labelled **1a**)	integrals (natural **1a**)	quotient of the integrals (enriched/natural)	[2-^{13}C] propionate enrichment [%]
C-1	173.0	0,6689	0,5189	1,2891	0,3
C-2	50.5	1,7354	1,0121	1,7147	0,8
C-3	26.7	1,1901	1,1311	1,0522	0,1
C-4	21.7	0,9864	1,0021	0,9843	0,0
C-5	47.1	1,0000	1,0000	1,0000	0,0
C-1´	177.0	0,4697	0,5556	0,8454	-0,2
C-2´	36.6	0,9025	0,9015	1,0011	0,0
C-3´	38.0	0,9381	0,9689	0,9682	0,0
C-4´	173.8	0,8729	0,6679	1,3069	0,3
C-5´	32.7	0,9927	0,9431	1,0526	0,1
C-6´	31.5	0,7636	0,6753	1,1308	0,1
C-7´	32.2	1,0172	1,0570	0,9623	0,0
C-8´	22.6	1,5603	1,2852	1,2141	0,2
C-9´	13.9	1,9738	2,0627	0,9569	0,0
C-1´´	26.12	1,3197	1,3584	0,9715	0,0
**C-2´´**	**40.7**	**15,4165**	**1,1430**	**13,4878**	**13,7**
C-3´´	208.1	0,9976	0,8218	1,2139	0,2
C-4´´	63.3	0,9906	0,9545	1,0378	0,0
C-5´´	35.67	1,8746	1,6042	1,1686	0,2
C-6´´	24.9	1,5678	1,4504	1,0809	0,1
C-7´´	11.2	1,6652	1,6215	1,0270	0,0
C-8´´	15.8	1,5881	1,6706	0,9506	-0,1
C-1´´´	171.3	0,4224	0,6679	0,6324	-0,4
C-2´´´	53.0	1,3127	1,3856	0,9474	-0,1
C-3´´´	34.2	n.d.[b]	n.d.[b]	n.d.[b]	n.d.[b]
C=O of COAc	169.8	0,7165	0,7568	0,9467	-0,1

[a] The integral of C-5 was set to 1.0000 for both ^{13}C NMR spectra because no incorporation was expected at this position; subsequently the integrals of the other carbon atoms were compared in the spectra of the labeled and the non-labeled compound **1a**; enrichment = enhancement of the signal x 1.1% - 1.1%; significant specific incorporations are indicated in red. [b] n.d. = not determined due to overlap with solvent signals.

Supplementary Table 7 ^{1}H and ^{13}C NMR data for His-matlystatin derivative **6**.

atom	δ_H[b]	δ_C[c]	^{1}H-^{1}H-COSY	^{1}H-^{13}C-HMBC	^{1}H-^{1}H-NOESY
1		172.2, qC			
2	5.21, dd (6.2, 2.5)	55.0, CH	3	1, 3, 4	3
3	2.20, m	26.7, CH_2	2, 4	2, 5	
	2.21, dd (13.9, 2.5)				
4	1.51, m	20.6, CH_2	3, 5		5
	1.71, m				
5	2.87, dd (12.4, 3.3)	42.9, CH_2	4		4
	4.57, dd (12.4)				
1´		173.5, qC			
2´	2.60, dd (4.9, 2.0)	41.2, CH	3´, 5´	1´, 3´, 5´	3´, 5´
3´	2.41, dd (16.1, 2.0)	34.3, CH_2	2´	1´, 2´, 4´	2´
	3.06, dd (16.1, 4.9)				
4´		169.1 qC			
5´	1.45, m	30.3, CH_2	2´, 6´	2´, 6´	
	1.55, m				
6´	1.32, m	27.8, CH_2	5´, 7´	5´, 7´	
	1.45, m				
7´	1.32, m	32.5, CH_2	6´, 8´	6´, 8´	
8´	1.32, m	23.5, CH_2	7´, 9´	7´, 9´	
9´	0.86, t (7.1)	14.3 CH_3	8´	7´, 8´	
1´´	4.25, dd (6.1, 6.1)	42.7, CH_2	2´´	2´´, 3´´, 5´´´, 6´´´	5´´´, 6´´´
2´´	3.03 dd (6.1, 6.1)	40.4, CH_2	1´´	1´´, 2´´	
3´´		207.9, qC			
4´´	4.32, d (6.0)	64.5, CH	5´´	1, 3´´, 5´´	
5´´	1.91, m	36.8, CH	6´´, 8´´		
6´´	1.13, m	25.5, CH_2	5´´, 7´´	5´´, 7´´, 8´´	
	1.25, m				
7´´	0.86, t (7.1)	11.7, CH_3	6´´	6´´	
8´´	0.88, d (6.9)	16.2, CH_3	5´´	5´´, 6´´	
1´´´		173.5, qC			
2´´´	3.79, dd (8.1, 4.0)	56.6, CH	3´´´	3´´´, 4´´´	
3´´´	2.95, dd (15.5, 8.1)	30.0, CH_2	2´´´	2´´´, 4´´´, 6´´´	
	3.17, dd (15.5, 4.0)				
4´´´		137.8, qC			
5´´´	7.64, br s	138.8, CH	6´´´	4´´´, 6´´´	1´´
6´´´	6.99, br s	118.9, CH	5´´´	5´´´	1´´

[a] Measured at 600 (^{1}H) and 150 MHz (^{13}C) in CD_3OD. [b] Coupling constants (*J*) are in parentheses and reported in Hz; s: singlet, d: doublet, m: multiplet, br: broad; chemical shifts are given in ppm. [c] Multiplicities were deduced from an edited HSQC experiment.

Supplementary Table 8 ^{1}H and ^{13}C NMR data for actinonin standard in CD_3OD.

**Position**	**δ_C**	**δ_H, mult. (*J* in Hz)**	**Position**	**δ_C**	**δ_H, mult. (*J* in Hz)**
1	170.8, C	-	11	173.1, C	-
2	36.5, CH_2	2.18, dd, 14.5, 7.9<br>2.34, dd, 14.5, 6.6	12	31.7, CH	2.04, m
3	43.7, CH	2.80, m	13*	19.0, CH_3	0.99, d, 6.9
4	177.2, C	-	14*	19.6, CH_3	0.96, d, 6.9
5	33.5, CH_2	1.39, m<br>1.55, m	15	60.9, CH	4.12, m
6	23.5, CH_2	1.29, m	16	28.0, CH_2	1.94, m
7	27.8, CH_2	1.25, m	17	25.1, CH_2	1.33, d, 5.6
8	32.8, CH_2	1.29, m	18	48.8, CH_2	3.57, ddd, 10.1, 7.1, 5.2<br>3.88, ddd, 10.1, 7.1, 7.1
9	14.3, CH_3	0.88, t, 7.0	19	63.3, CH_2	3.50, dd, 10.8, 6.4<br>3.67, dd, 10.8, 4.3
10	58.2, CH	4.38, d, 8.6			

*Signal may be interchanged.
