# Supplementary material for: Warhead biosynthesis and the origin of structural diversity in hydroxamate metalloproteinase inhibitors
Source: Nat Commun. 2017 Dec 6;8:1965. doi: 10.1038/s41467-017-01975-6 (PMC5719088; doi:10.1038/s41467-017-01975-6)
Supplement: Supplementary file 3 — Description of Additional Supplementary Files [file 41467_2017_1975_MOESM3_ESM.pdf]

**Descriptions of Additional Supplementary Files**

File Name: Supplementary Dataset 1
Description: MS network cytoscape file
